# Supplementary material for: The topography of corticopontine projections is controlled by postmitotic expression of the area-mapping gene Nr2f1
Source: Development. 2022 Mar 9;149(5):dev200026. doi: 10.1242/dev.200026 (PMC8959144; doi:10.1242/dev.200026)
Supplement: Supplementary information [file develop-149-200026-s1.pdf]

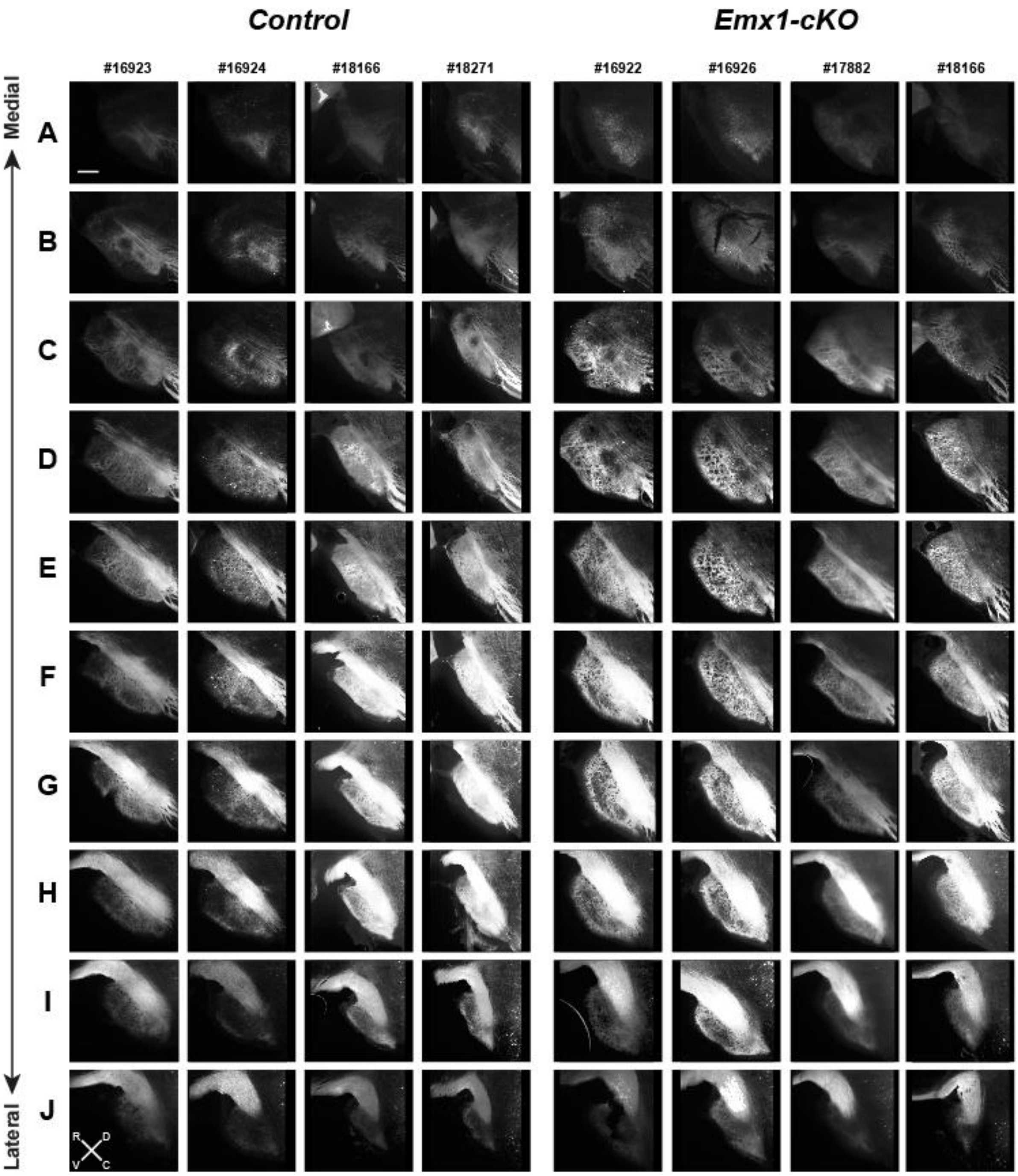

Fig. S1. Overview of YFP-expression in *Emx1-cKO* mice and controls

Fluorescence microscopy images of the pontine nuclei in sagittal sections from 4 control and 4 *Emx1-cKO* mice. Columns show images from one animal, with sections from corresponding levels from medial to lateral sorted from top to bottom (rows A-J). The intensity levels of the images have been normalized. Signal expression in *Emx1-cKO* mice is more widespread and more diffusely distributed in the pontine nuclei, relative to controls. Scale bar, 200µm.

Development • Supplementary information

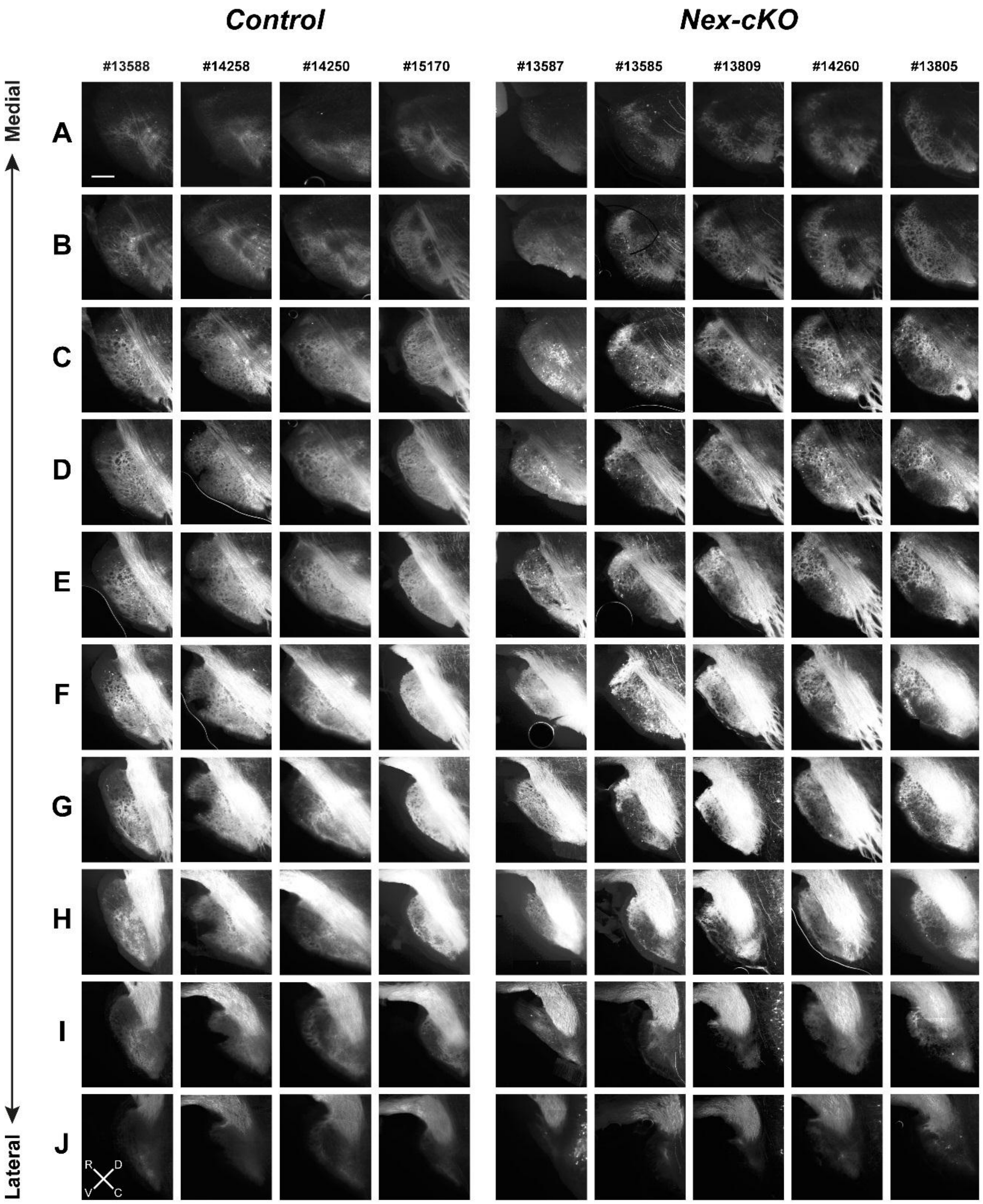

Fig. S2. Overview of YFP-expression in *Nex-cKO* mice and controls

Fluorescence microscopy images of the pontine nuclei in sagittal sections from 4 control and 4 *Nex-cKO* mice. Columns show images from one animal, with sections from corresponding levels from medial to lateral sorted from top to bottom (rows A-J). The intensity levels of the images have been normalized. Signal expression in *Nex-cKO* mice is reduced or absent in the central core region of the pontine nuclei, relative to controls. Scale bar, 200µm.

Development • Supplementary information

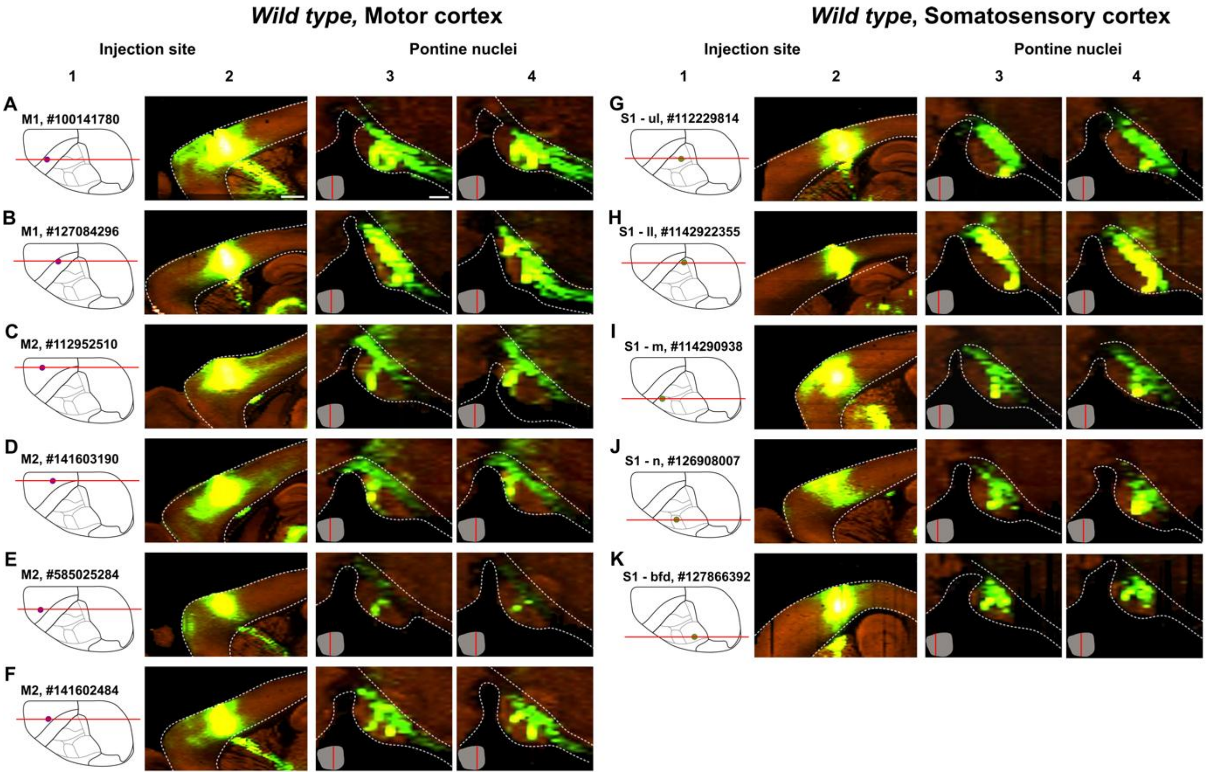

Fig. S3. Overview of tract-tracing experiments in wild-type mice

(**A-K**) Sagittally-oriented fluorescence microscopy images showing all wild-type C57BL/6J mouse tract-tracing data captured from the online image viewer of the Allen Mouse Brain Connectivity Atlas. **Column 1** shows dorsal view diagrams of the cerebral cortex indicating the position of the tracer injection site as a red dot, and a red line indicating the location of the sagittal images in **column 2** that show injection site centres. Letters and numbers indicate injected cortical area and ID numbers. **Columns 3 and 4** show fluorescence microscopy images of sagittal sections through the pontine nuclei, showing representative corticopontine labelling at two mediolateral levels as indicated with red lines on the inset ventral view diagram of the pontine nuclei. Abbreviations, bfd, barrel field; M1, primary motor cortex; M2, secondary motor cortex; ll, lower limb; m, mouth; n, nose; ul, upper limb. Scale bars, 1mm (column 2), 200µm (columns 3, 4).

Development • Supplementary information

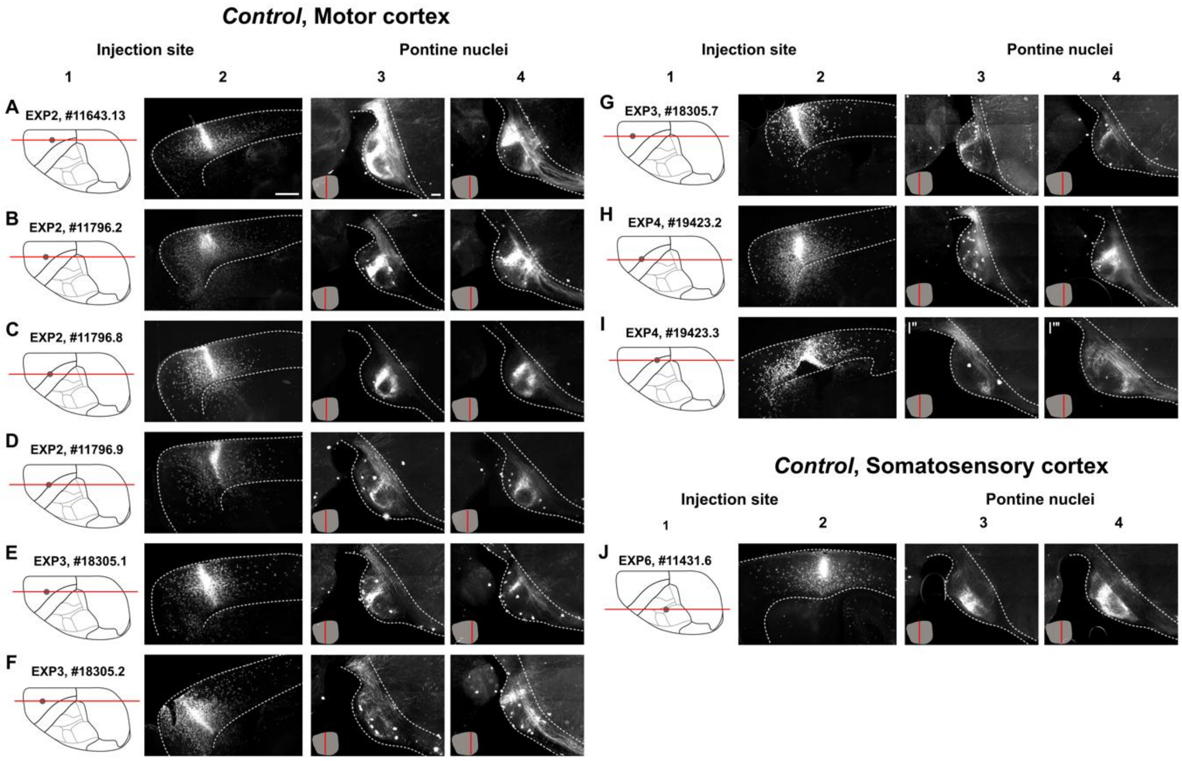

Fig. S4. Overview of tract-tracing experiments in control mice

(**A-J**) Sagittally-oriented fluorescence microscopy images showing all tract-tracing data conducted in control animals. **Column 1** shows dorsal view diagrams of the cerebral cortex indicating the position of the tracer injection site as a red dot, and a red line indicating the location of the sagittal images in **column 2** that show injection site centres. Letters and numbers indicate injected cortical area and ID numbers. **Columns 3 and 4** show fluorescence microscopy images of sagittal sections through the pontine nuclei, showing representative corticopontine labelling at two mediolateral levels as indicated with red lines on the inset ventral view diagram of the pontine nuclei. Scale bars, 1mm (column 2), 200µm (columns 3, 4).

Development • Supplementary information

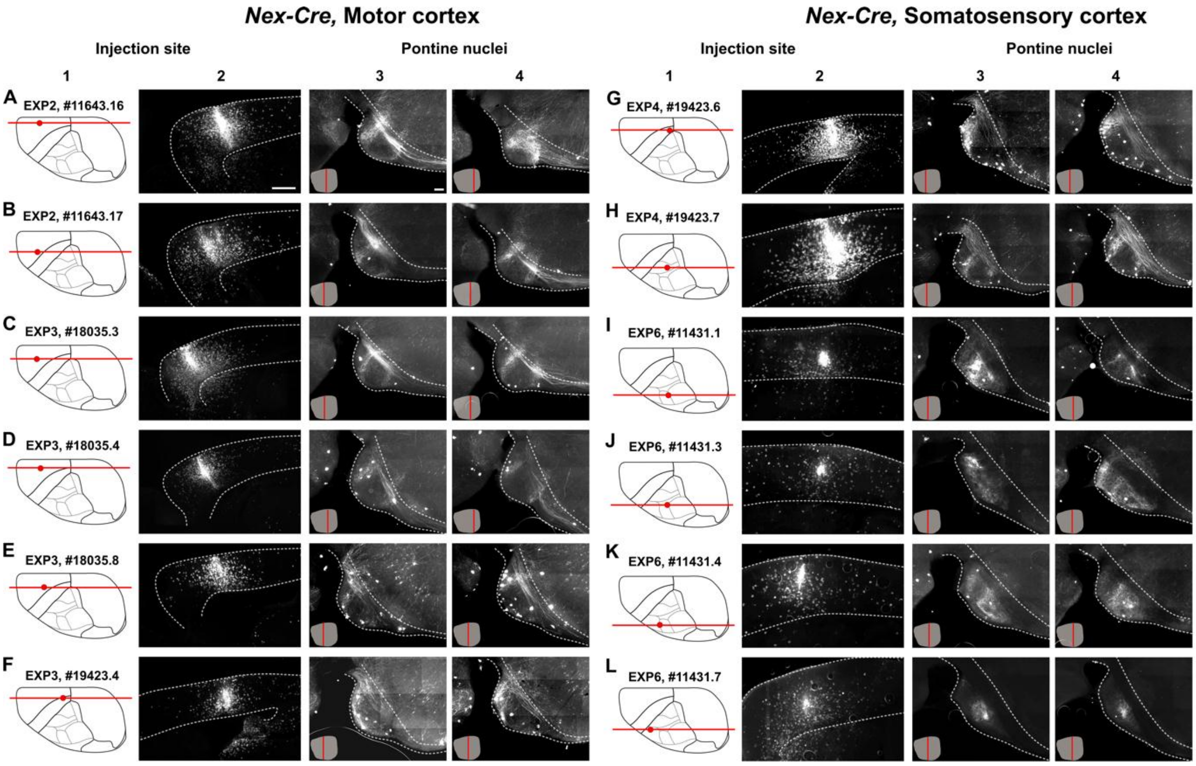

Fig. S5. Overview of tract-tracing experiments in *Nex-cKO* mice

(**A-L**) Sagittally-oriented fluorescence microscopy images showing all tract-tracing data conducted in *Nex-cKO* animals. **Column 1** shows dorsal view diagrams of the cerebral cortex indicating the position of the tracer injection site as a red dot, and a red line indicating the location of the sagittal images in **column 2** that show injection site centres. Letters and numbers indicate injected cortical area and ID numbers. **Columns 3 and 4** show fluorescence microscopy images of sagittal sections through the pontine nuclei, showing representative corticopontine labelling at two mediolateral levels as indicated with red lines on the inset ventral view diagram of the pontine nuclei. Scale bars, 1mm (column 2), 200µm (columns 3, 4).

Table S1. Overview of wild-type experiments from the Allen Mouse Brain Connectivity database

Allen Mouse Brain Connectivity database					
**Experiment number #**	**Sex**	**Age (±2)**	**Genotype**	**Injection site**	**Shown in**
100141780	Male	P56	C57BL/6J	Primary motor cortex	Fig. 2, 6A-F
114290938	male	P56	C57BL/6J	Primary somatosensory cortex, mouth region	Fig. 2A-P, 7A and 7H
112229814	male	P56	C57BL/6J	Primary somatosensory cortex, upper limb region	Fig. 2, 7B and 7F
112952510	male	P56	C57BL/6J	Secondary motor cortex	Fig. 2
114292355	male	P56	C57BL/6J	Primary somatosensory cortex, lower limb region	Fig. 2, 7A and 7D
126908007	male	P56	C57BL/6J	Primary somatosensory cortex, nose region	Fig. 2, 7A and 7G
127084296	male	P56	C57BL/6J	Secondary motor cortex	Fig. 2
127866392	male	P56	C57BL/6J	Primary somatosensory cortex, barrel field region	Fig. 2
141602484	male	P56	C57BL/6J	Secondary motor cortex	Fig. 2
141603190	male	P56	C57BL/6J	Secondary motor cortex	Fig. 2
585025284	male	P56	C57BL/6J	Secondary motor cortex	Fig. 2

Development • Supplementary information

Table S2. Overview of *Emx1-cKO* and *Nex-cKO* mice

Adult					
**Exp. #**	**Animal #**	**Sex**	**Age**	**Genotype**	**Shown in**
1	13588	female	P33	*Thy1-eYFP*$^{T/+}$; *Nr2f1*$^{fl/fl}$	Fig. 3, 4, 5 and Suppl. Fig.1, 2
1	13587	female	P33	*Thy1-eYFP*$^{T/+}$; *Nr2f1*$^{fl/fl}$; *Nex-Cre*	Fig. 3, 4, 5 and Suppl. Fig.1, 2
2	13585	male	P62	*Thy1-eYFP*$^{T/+}$; *Nr2f1*$^{fl/fl}$; *Nex-Cre*	Fig. 3, 4, 5 and Suppl. Fig.1, 2
3	13809	male	P57	*Thy1-eYFP*$^{T/+}$; *Nr2f1*$^{fl/fl}$; *Nex-Cre*	Fig. 3, 4, 5 and Suppl. Fig.1, 2
4	14258	male	P57	*Thy1-eYFP*$^{T/+}$; *Nr2f1*$^{fl/fl}$	Fig. 3, 4, 5 and Suppl. Fig.1, 2
4	14260	male	P57	*Thy1-eYFP*$^{T/+}$; *Nr2f1*$^{fl/fl}$; *Nex-Cre*	Fig. 3, 4, 5 and Suppl. Fig.1, 2
5	13805	male	P55	*Thy1-eYFP*$^{T/+}$; *Nr2f1*$^{fl/fl}$; *Nex-Cre*	Fig. 3, 4, 5 and Suppl. Fig.1, 2
5	14250	male	P72	*Thy1-eYFP*$^{T/+}$; *Nr2f1*$^{fl/fl}$	Fig. 3, 4, 5 and Suppl. Fig.1, 2
5	15170	male	P75	*Thy1-eYFP*$^{T/+}$; *Nr2f1*$^{fl/fl}$	Fig. 3, 4, 5 and Suppl. Fig.1, 2
6	16922	male	P76	*Thy1-eYFP*$^{T/+}$; *Nr2f1*$^{fl/fl}$; *Emx1-Cre*	Fig. 3, 4, 5 and Suppl. Fig.1, 2
6	16923	male	P76	*Thy1-eYFP*$^{T/+}$; *Nr2f1*$^{fl/fl}$	Fig. 3, 4, 5 and Suppl. Fig.1, 2
6	16924	male	P76	*Thy1-eYFP*$^{T/+}$; *Nr2f1*$^{fl/fl}$	Fig. 3, 4, 5 and Suppl. Fig.1, 2
6	16926	male	P76	*Thy1-eYFP*$^{T/+}$; *Nr2f1*$^{fl/fl}$; *Emx1-Cre*	Fig. 3, 4, 5 and Suppl. Fig.1, 2
7	17882	male	P72	*Thy1-eYFP*$^{T/+}$; *Nr2f1*$^{fl/fl}$; *Emx1-Cre*	Fig. 3, 4, 5 and Suppl. Fig.1, 2
8	18046	female	P109	*Thy1-eYFP*$^{T/+}$; *Nr2f1*$^{fl/fl}$; *Emx1-Cre*	Fig. 3, 4, 5 and Suppl. Fig.1, 2
8	18166	female	P98	*Thy1-eYFP*$^{T/+}$; *Nr2f1*$^{fl/fl}$	Fig. 3, 4, 5 and Suppl. Fig.1, 2
8	18271	female	P87	*Thy1-eYFP*$^{T/+}$; *Nr2f1*$^{fl/fl}$	Fig. 3, 4, 5 and Suppl. Fig.1, 2
9	19606	female	P86	*Thy1-eYFP*$^{T/+}$; *Nr2f1*$^{fl/fl}$; *Emx1-Cre*	Fig. 4
9	19607	female	P86	*Thy1-eYFP*$^{T/+}$; *Nr2f1*$^{fl/fl}$; *Emx1-Cre*	Fig. 4

Development • Supplementary information

Table S3. Overview of tract tracing experiments in *Nex-cKO* and *Ctrl* mice

**P21 – unilateral CST tracing**			
Tracer injected in motor cortex			
**Experiment #**	**Animal #**	**Genotype**	**Shown in**
2	11643_13	*Ctrl*	Fig. 6A and 7B
2	11643_16	*Nex-cKO*	Suppl. Fig. 3
2	11643_17	*Nex-cKO*	Fig. 6A and 6F
2	11796_2	*Ctrl*	Suppl. Fig. 3
2	11796_8	*Ctrl*	Suppl. Fig. 3
2	11796_9	*Ctrl*	Suppl. Fig. 3
3	18035_1	*Ctrl*	Fig. 6A and 6F
3	18035_2	*Ctrl*	Fig. 6A and 6E
3	18035_7	*Ctrl*	Suppl. Fig. 3
3	18035_3	*Nex-cKO*	Fig. 6A and 6E
3	18035_4	*Nex-cKO*	Suppl. Fig. 3
3	18035_8	*Nex-cKO*	Fig. 6A and 76D
4	19423_2	*Ctrl*	Suppl. Fig. 3
4	19423_3	*Ctrl*	Suppl. Fig. 3
4	19423_4	*Nex-cKO*	Suppl. Fig. 3
4	19423_5	*Nex-cKO*	Suppl. Fig. 3
Tracer injected in somatosensory cortex			
4	19423_6	*Nex-cKO*	Fig. 7A and 7D
4	19423_7	*Nex-cKO*	Fig. 7A and 7E
6	11431_1	*Nex-cKO*	Fig. 7A and 7G
6	11431_3	*Nex-cKO*	Fig. 7A and 7H
6	11431_4	*Nex-cKO*	Fig. 7A and 7F
6	11431_6	*Ctrl*	Fig. 7A and 7F
6	11431_7	*Nex-cKO*	Fig. 7A and 7H

Development • Supplementary information

Table S4. Overview of quantitative results.

Highlighted in blue, comparisons that produced statistically significant P-values. P-values are calculated by 2way ANOVA test (**Figures 3F,G and 4E-F**), or ordinary one-way ANOVA test (**Figures 4G-I**).

Figure 3B”-D”– Cortical distribution of YFP-H positive cells (% values)							
**Area**	**Hypothesis**	**Mean 1**	**Mean 2**	**Mean diff.**	**95,00% CI of diff**	**Summary**	**Adjusted P.**
**PFC**	*Ctrl vs. Nex-cKO*	4.833	1.95	2.883	-3.655 to 9.421	ns	0.5437
	Ctrl vs. Emx1-cKO	4.833	3.15	1.683	-4.855 to 8.221	ns	0.8112
	Nex-cKO vs. Emx1-cKO	1.95	3.15	-1.2	-8.362 to 5.962	ns	0.9150
**M**	*Ctrl vs. Nex-cKO*	46.87	24.69	22.18	15.64 to 28.71	****	<0.0001
	Ctrl vs. Emx1-cKO	46.87	27.08	19.78	13.25 to 26.32	****	<0.0001
	Nex-cKO vs. Emx1-cKO	24.69	27.08	-2.393	-9.555 to 4.769	ns	0.7036
**S**	*Ctrl vs. Nex-cKO*	23.25	27.56	-4.312	-10.85 to 2.226	ns	0.2608
	Ctrl vs. Emx1-cKO	23.25	23.42	-0.1744	-6.712 to 6.363	ns	0.9977
	Nex-cKO vs. Emx1-cKO	27.56	23.42	4.137	-3.024 to 11.3	ns	0.3545
**A**	*Ctrl vs. Nex-cKO*	5.671	8.786	-3.115	-9.653 to 3.423	ns	0.4917
	Ctrl vs. Emx1-cKO	5.671	5.947	-0.2756	-6.813 to 6.262	ns	0.9944
	Nex-cKO vs. Emx1-cKO	8.786	5.947	2.839	-4.323 to 10	ns	0.6105
**V**	*Ctrl vs. Nex-cKO*	10.13	21.5	-11.36	-17.9 to -4.825	***	0.0003
	Ctrl vs. Emx1-cKO	10.13	21.35	-11.22	-17.76 to -4.681	***	0.0003
	Nex-cKO vs. Emx1-cKO	21.5	21.35	0.1434	-7.018 to 7.305	ns	0.9987
**RSC**	*Ctrl vs. Nex-cKO*	9.184	15.5	-6.318	-12.86 to 0.2199	ns	0.0604
	Ctrl vs. Emx1-cKO	9.184	19.01	-9.821	-16.36 to -3.284	**	0.0017
	Nex-cKO vs. Emx1-cKO	15.5	19.01	-3.503	-10.67 to 3.658	ns	0.4734
**Figure 3E– Cortical distribution of YFP-H positive cells (#of cells/slides)**							
**Area**	**Hypothesis**	**Mean 1**	**Mean 2**	**Mean diff.**	**95,00% CI of diff**	**Summary**	**Adjusted P.**
**PFC**	*Ctrl vs. Nex-cKO*	*33.06*	*6.42*	*26.64*	*-52.67 to 105.9*	*ns*	*0.5048*
	Ctrl vs. Emx1-cKO	*33.06*	*40.33*	*-7.269*	*-86.57 to 72.03*	*ns*	*0.8553*
	Nex-cKO vs. Emx1-cKO	*6.42*	*40.33*	*-33.91*	*-120.8 to 52.96*	*ns*	*0.4386*
**M**	*Ctrl vs. Nex-cKO*	*324.5*	*91.87*	*232.6*	*153.3 to 311.9*	****	*<0.0001*
	Ctrl vs. Emx1-cKO	*324.5*	*301.7*	*22.78*	*-56.52 to 102.1*	*ns*	*0.5682*
	Nex-cKO vs. Emx1-cKO	*91.87*	*301.7*	*-209.9*	*-296.7 to -123*	****	*<0.0001*
**S**	*Ctrl vs. Nex-cKO*	*163.2*	*105.2*	*57.98*	*-21.32 to 137.3*	*ns*	*0.1491*
	Ctrl vs. Emx1-cKO	*163.2*	*257.8*	*-94.58*	*-173.9 to -15.28*	*	*0.0202*
	Nex-cKO vs. Emx1-cKO	*105.2*	*257.8*	*-152.6*	*-239.4 to -65.69*	***	*0.0008*
**A**	*Ctrl vs. Nex-cKO*	*38.09*	*32.77*	*5.32*	*-73.98 to 84.62*	*ns*	*0.8939*
	Ctrl vs. Emx1-cKO	*38.09*	*61.63*	*-23.53*	*-102.8 to 55.77*	*ns*	*0.5555*
	Nex-cKO vs. Emx1-cKO	*32.77*	*61.63*	*-28.85*	*-115.7 to 58.02*	*ns*	*0.5095*

**V**	*Ctrl vs. Nex-cKO*	*81.24*	*81.07*	*0.1681*	*-79.13 to 79.47*	*ns*	*0.9966*
	Ctrl vs. Emx1-cKO	*81.24*	*228*	*-146.8*	*-226.1 to -67.45*	*****	*0.0004*
	Nex-cKO vs. Emx1-cKO	*81.07*	*228*	*-146.9*	*-233.8 to -60.05*	****	*0.0012*
**RSC**	*Ctrl vs. Nex-cKO*	*64.39*	*58.41*	*5.974*	*-73.33 to 85.28*	*ns*	*0.8809*
	Ctrl vs. Emx1-cKO	*64.39*	*204.1*	*-139.8*	*-219.1 to -60.46*	*****	*0.0008*
	Nex-cKO vs. Emx1-cKO	*58.41*	*204.1*	*-145.7*	*-232.6 to -58.86*	****	*0.0013*
**Figure 3F– Distribution of YFP-H positive cells among M and S areas (% values)**							
**Area**	**Hypothesis**	**Mean 1**	**Mean 2**	**Mean diff.**	**95,00% CI of diff**	**Summary**	**Adjusted P.**
**M**	*Ctrl vs. Nex-cKO*	*66.85*	*47.35*	*19.5*	*7.864 to 31.14*	****	*0.0021*
	Ctrl vs. Emx1-cKO	*66.85*	*53.43*	*13.43*	*1.79 to 25.07*	***	*0.0257*
	Nex-cKO vs. Emx1-cKO	*47.35*	*53.43*	*-6.074*	*-18.82 to 6.676*	*ns*	*0.3339*
**S**	*Ctrl vs. Nex-cKO*	*33.15*	*52.65*	*-19.5*	*-31.14 to -7.864*	****	*0.0021*
	Ctrl vs. Emx1-cKO	*33.15*	*46.57*	*-13.43*	*-25.07 to -1.79*	***	*0.0257*
	Nex-cKO vs. Emx1-cKO	*52.65*	*46.57*	*6.074*	*-6.676 to 18.82*	*ns*	*0.3339*
**Figure 4E – Rostral LFP diameter**							
**Section**	**Hypothesis**	**Mean 1**	**Mean 2**	**Mean diff.**	**95,00% CI of diff**	**Summary**	**Adjusted P.**
**1**	*Ctrl vs. Nex-cKO*	0	0	0	-116.9 to 116.9	ns	>0.9999
	Ctrl vs. Emx1-cKO	0	0	0	-116.9 to 116.9	ns	>0.9999
	Nex-cKO vs. Emx1-cKO	0	0	0	-124.9 to 124.9	ns	>0.9999
**2**	*Ctrl vs. Nex-cKO*	0	28.29	-28.29	-145.2 to 88.57	ns	0.836
	Ctrl vs. Emx1-cKO	0	0	0	-116.9 to 116.9	ns	>0.9999
	Nex-cKO vs. Emx1-cKO	28.29	0	28.29	-96.64 to 153.2	ns	0.8549
**3**	*Ctrl vs. Nex-cKO*	0	132.7	-132.7	-249.6 to -15.84	*	0.0215
	Ctrl vs. Emx1-cKO	0	0	0	-116.9 to 116.9	ns	>0.9999
	Nex-cKO vs. Emx1-cKO	132.7	0	132.7	7.775 to 257.6	*	0.0343
**4**	*Ctrl vs. Nex-cKO*	0	179.6	-179.6	-296.5 to -62.77	**	0.001
	Ctrl vs. Emx1-cKO	0	0	0	-116.9 to 116.9	ns	>0.9999
	Nex-cKO vs. Emx1-cKO	179.6	0	179.6	54.71 to 304.6	**	0.0023
**5**	*Ctrl vs. Nex-cKO*	0	175.9	-175.9	-292.8 to -59.04	**	0.0013
	Ctrl vs. Emx1-cKO	0	55.34	-55.34	-172.2 to 61.52	ns	0.5052
	Nex-cKO vs. Emx1-cKO	175.9	55.34	120.6	-4.369 to 245.5	ns	0.0612
**6**	*Ctrl vs. Nex-cKO*	24.35	169.6	-145.2	-262.1 to -28.37	*	0.0103
	Ctrl vs. Emx1-cKO	24.35	115	-90.67	-207.5 to 26.2	ns	0.1624
	Nex-cKO vs. Emx1-cKO	169.6	115	54.57	-70.37 to 179.5	ns	0.5592
**7**	*Ctrl vs. Nex-cKO*	117	231.4	-114.4	-231.3 to 2.467	ns	0.0565
	Ctrl vs. Emx1-cKO	117	234.6	-117.7	-234.5 to -0.8201	*	0.048
	Nex-cKO vs. Emx1-	231.4	234.6	-3.287	-128.2 to 121.6	ns	0.9979

Development • Supplementary information

	cKO						
**8**	*Ctrl vs. Nex-cKO*	241.7	187.5	54.13	-66.26 to 174.5	ns	0.5401
	Ctrl vs. Emx1-cKO	241.7	293	-51.33	-171.7 to 69.06	ns	0.5746
	Nex-cKO vs. Emx1-cKO	187.5	293	-105.5	-230.4 to 19.47	ns	0.1168
**9**	*Ctrl vs. Nex-cKO*	316.6	267.9	48.71	-68.15 to 165.6	ns	0.5888
	Ctrl vs. Emx1-cKO	316.6	250.1	66.55	-56.81 to 189.9	ns	0.4128
	Nex-cKO vs. Emx1-cKO	267.9	250.1	17.83	-113.2 to 148.9	ns	0.9449
**10**	*Ctrl vs. Nex-cKO*	318.9	194.4	124.6	7.715 to 241.4	*	0.0335
	Ctrl vs. Emx1-cKO	318.9	205.8	113.1	-3.738 to 230	ns	0.0602
	Nex-cKO vs. Emx1-cKO	194.4	205.8	-11.45	-136.4 to 113.5	ns	0.9746
**11**	*Ctrl vs. Nex-cKO*	231.1	88.06	143.1	-163 to 449.1	ns	0.514
	Ctrl vs. Emx1-cKO	231.1	64.04	167.1	-82.79 to 416.9	ns	0.258
	Nex-cKO vs. Emx1-cKO	88.06	64.04	24.02	-225.8 to 273.9	ns	0.9721
**12**	*Ctrl vs. Nex-cKO*	276.1	179.6	96.5	-26.86 to 219.9	ns	0.1576
	Ctrl vs. Emx1-cKO	276.1	136.5	139.6	16.23 to 263	*	0.022
	Nex-cKO vs. Emx1-cKO	179.6	136.5	43.09	-93.77 to 179.9	ns	0.7389
**13**	*Ctrl vs. Nex-cKO*	201.9	180.5	21.33	-243.7 to 286.3	ns	0.9804
	Ctrl vs. Emx1-cKO	201.9	44.45	157.4	-40.14 to 354.9	ns	0.1471
	Nex-cKO vs. Emx1-cKO	180.5	44.45	136.1	-113.8 to 385.9	ns	0.4059
**14**	*Ctrl vs. Nex-cKO*	218.4	114.6	103.8	-19.52 to 227.2	ns	0.1182
	Ctrl vs. Emx1-cKO	218.4	95.41	123	-0.3344 to 246.4	ns	0.0508
	Nex-cKO vs. Emx1-cKO	114.6	95.41	19.19	-117.7 to 156	ns	0.9416
**15**	*Ctrl vs. Nex-cKO*	154.7	59.11	95.61	-21.25 to 212.5	ns	0.1328
	Ctrl vs. Emx1-cKO	154.7	51.17	103.6	-13.31 to 220.4	ns	0.0942
	Nex-cKO vs. Emx1-cKO	59.11	51.17	7.94	-117 to 132.9	ns	0.9877
**16**	*Ctrl vs. Nex-cKO*	101.6	16.85	84.78	-32.09 to 201.6	ns	0.2035
	Ctrl vs. Emx1-cKO	101.6	18.75	82.87	-33.99 to 199.7	ns	0.2183
	Nex-cKO vs. Emx1-cKO	16.85	18.75	-1.908	-126.8 to 123	ns	0.9993
**17**	*Ctrl vs. Nex-cKO*	60.94	0	60.94	-55.93 to 177.8	ns	0.4374
	Ctrl vs. Emx1-cKO	60.94	0	60.94	-55.93 to 177.8	ns	0.4374
	Nex-cKO vs. Emx1-cKO	0	0	0	-124.9 to 124.9	ns	>0.9999
**18**	*Ctrl vs. Nex-cKO*	28.85	0	28.85	-88.02 to 145.7	ns	0.8301
	Ctrl vs. Emx1-cKO	28.85	0	28.85	-88.02 to 145.7	ns	0.8301
	Nex-cKO vs. Emx1-cKO	0	0	0	-124.9 to 124.9	ns	>0.9999
**19**	*Ctrl vs. Nex-cKO*	0	0	0	-112 to 112	ns	>0.9999
	Ctrl vs. Emx1-cKO	0	0	0	-116.9 to 116.9	ns	>0.9999
	Nex-cKO vs. Emx1-cKO	0	0	0	-120.4 to 120.4	ns	>0.9999
**Figure 4F – Caudal LFP diameter**							
**Section**	**Hypothesis**	**Mean 1**	**Mean 2**	**Mean diff.**	**95,00% CI of diff**	**Summary**	**Adjusted P.**
**1**	*Ctrl vs. Nex-cKO*	0	0	0	-88.51 to 88.51	ns	>0.9999
	Ctrl vs. Emx1-cKO	0	0	0	-88.51 to 88.51	ns	>0.9999
	Nex-cKO vs. Emx1-	0	0	0	-94.62 to 94.62	ns	>0.9999

Development • Supplementary information

	cKO						
**2**	*Ctrl vs. Nex-cKO*	0	46.89	-46.89	-135.4 to 41.62	ns	0.4252
	Ctrl vs. Emx1-cKO	0	0	0	-88.51 to 88.51	ns	>0.9999
	Nex-cKO vs. Emx1-cKO	46.89	0	46.89	-47.73 to 141.5	ns	0.4728
**3**	*Ctrl vs. Nex-cKO*	0	98.1	-98.1	-186.6 to -9.584	*	0.0257
	Ctrl vs. Emx1-cKO	0	0	0	-88.51 to 88.51	ns	>0.9999
	Nex-cKO vs. Emx1-cKO	98.1	0	98.1	3.473 to 192.7	*	0.0402
**4**	*Ctrl vs. Nex-cKO*	0	126.2	-126.2	-214.7 to -37.72	**	0.0026
	Ctrl vs. Emx1-cKO	0	0	0	-88.51 to 88.51	ns	>0.9999
	Nex-cKO vs. Emx1-cKO	126.2	0	126.2	31.6 to 220.9	**	0.0053
**5**	*Ctrl vs. Nex-cKO*	18.5	111	-92.51	-181 to -3.999	*	0.0382
	Ctrl vs. Emx1-cKO	18.5	13.31	5.196	-83.32 to 93.71	ns	0.9895
	Nex-cKO vs. Emx1-cKO	111	13.31	97.71	3.084 to 192.3	*	0.0412
**6**	*Ctrl vs. Nex-cKO*	69.28	128.1	-58.82	-147.3 to 29.69	ns	0.2617
	Ctrl vs. Emx1-cKO	69.28	97.47	-28.19	-116.7 to 60.32	ns	0.7331
	Nex-cKO vs. Emx1-cKO	128.1	97.47	30.63	-63.99 to 125.3	ns	0.7256
**7**	*Ctrl vs. Nex-cKO*	107.5	176	-68.56	-300.3 to 163.2	ns	0.765
	Ctrl vs. Emx1-cKO	107.5	152.1	-44.66	-227.9 to 138.6	ns	0.8336
	Nex-cKO vs. Emx1-cKO	176	152.1	23.9	-159.3 to 207.1	ns	0.9491
**8**	*Ctrl vs. Nex-cKO*	175.2	237.2	-61.95	-157.9 to 34.02	ns	0.2821
	Ctrl vs. Emx1-cKO	175.2	154.5	20.69	-70.49 to 111.9	ns	0.8541
	Nex-cKO vs. Emx1-cKO	237.2	154.5	82.63	-16.61 to 181.9	ns	0.1235
**9**	*Ctrl vs. Nex-cKO*	214.5	138.2	76.34	-12.17 to 164.9	ns	0.1063
	Ctrl vs. Emx1-cKO	214.5	197.7	16.82	-71.69 to 105.3	ns	0.8952
	Nex-cKO vs. Emx1-cKO	138.2	197.7	-59.52	-154.1 to 35.1	ns	0.3005
**10**	*Ctrl vs. Nex-cKO*	209.6	115.1	94.49	5.983 to 183	*	0.0333
	Ctrl vs. Emx1-cKO	209.6	132	77.6	-10.91 to 166.1	ns	0.0988
	Nex-cKO vs. Emx1-cKO	115.1	132	-16.89	-111.5 to 77.73	ns	0.9069
**11**	*Ctrl vs. Nex-cKO*	232.5	99.13	133.4	44.9 to 221.9	**	0.0013
	Ctrl vs. Emx1-cKO	232.5	116.2	116.4	27.87 to 204.9	**	0.0061
	Nex-cKO vs. Emx1-cKO	99.13	116.2	-17.03	-111.7 to 77.59	ns	0.9055
**12**	*Ctrl vs. Nex-cKO*	186.4	127.8	58.65	-34.78 to 152.1	ns	0.302
	Ctrl vs. Emx1-cKO	186.4	26.38	160.1	71.56 to 248.6	****	<0.0001
	Nex-cKO vs. Emx1-cKO	127.8	26.38	101.4	2.179 to 200.7	*	0.0439
**13**	*Ctrl vs. Nex-cKO*	94.47	0	94.47	-137.3 to 326.2	ns	0.6019
	Ctrl vs. Emx1-cKO	94.47	13.38	81.09	-95.93 to 258.1	ns	0.527
	Nex-cKO vs. Emx1-cKO	0	13.38	-13.38	-190.4 to 163.6	ns	0.9826
**14**	*Ctrl vs. Nex-cKO*	58.1	0	58.1	-33.08 to 149.3	ns	0.2913
	Ctrl vs. Emx1-cKO	58.1	0	58.1	-33.08 to 149.3	ns	0.2913
	Nex-cKO vs. Emx1-cKO	0	0	0	-94.62 to 94.62	ns	>0.9999
**15**	*Ctrl vs. Nex-cKO*	0	0	0	-84.82 to 84.82	ns	>0.9999

Development • Supplementary information

	Ctrl vs. Emx1-cKO	0	0	0	-88.51 to 88.51	ns	>0.9999
	Nex-cKO vs. Emx1-cKO	0	0	0	-91.18 to 91.18	ns	>0.9999
**Figure 4G – Rostral LFP area**							
	**Hypothesis**	**Mean 1**	**Mean 2**	**Mean diff.**	**95,00% CI of diff**	**Summary**	**Adjusted P.**
	Ctrl vs. Nex-cKO	2414	2258	156.4	-570.3 to 883	ns	0.8257
	Ctrl vs. Emx1-cKO	2414	1725	689.2	-37.42 to 1416	ns	0.0637
**Figure 4H – Caudal LFP area**							
	**Hypothesis**	**Mean 1**	**Mean 2**	**Mean diff.**	**95,00% CI of diff**	**Summary**	**Adjusted P.**
	Ctrl vs. Nex-cKO	1403	1427	-24.29	-261.8 to 213.2	ns	0.9566
	Ctrl vs. Emx1-cKO	1403	884.1	518.8	281.3 to 756.3	***	0.0001
**Figure 4I – Fasciculation Index**							
**Region**	**Hypothesis**	**Mean 1**	**Mean 2**	**Mean diff.**	**95,00% CI of diff**	**Summary**	**Adjusted P.**
**250 µm**	*Ctrl vs. Nex-cKO*	0.6528	0.5493	0.1035	0.01185 to 0.1951	*	0.0216
	Ctrl vs. Emx1-cKO	0.6528	0.5595	0.09326	0.001658 to 0.1849	*	0.0446
**500 µm**	*Ctrl vs. Nex-cKO*	0.674	0.5771	0.0969	0.00254 to 0.1913	*	0.0422
	Ctrl vs. Emx1-cKO	0.674	0.5146	0.1594	0.06502 to 0.2537	***	0.0004

Table S5. List of primary and secondary antibodies used in this study.

Antigen	Provider	Catalog #	Species	Working dilution
GFP	Abcam	Ab13970	Ck	1:500
RFP	Abcam	Ab 124754	Rb	1:500
Ck IgY - AF 488	Thermo Fisher	A11039	Gt	1:500
Rb IgG - AF 555	Thermo Fisher	A21428	Gt	1:500

Table S6. Raw data and statistical analyses related to Table S4.

Click here to download Table S6
