# Supplementary material for: The topography of corticopontine projections is controlled by postmitotic expression of the area-mapping gene Nr2f1
Source: Development. 2022 Mar 9;149(5):dev200026. doi: 10.1242/dev.200026 (PMC8959144; doi:10.1242/dev.200026)
Supplement: Reviewer comments [file develop-149-200026_review_history.pdf]

The topography of corticopontine projections is controlled by postmitotic expression of the area-mapping gene *Nr2f1*

Chiara Tocco, Martin Øvsthus, Jan G. Bjaalie, Trygve B. Leergaard and Michèle Studer
DOI: 10.1242/dev.200026

Editor: James Briscoe

Review timeline

Submission to Review Commons:	11 May 2021
Submission to Development:	16 July 2021
Editorial decision:	28 July 2021
First revision received:	10 September 2021
Editorial decision:	4 October 2021
Second revision received:	3 November 2021
Editorial decision:	10 December 2021
Third revision received:	16 December 2021
Accepted:	20 December 2021

Reviewer 1

Evidence, reproducibility and clarity

Tocco et al examine the changes in the topographic organization of corticopontine projections and infer the ratio of corticopontine versus corticospinal projections from motor and somatosensory cortex after knocking out the expression of Nr2f1 at two developmental timepoints. They conclude that Nr2f1 acts as a molecular determinant of the ratio between corticopontine and corticospinal projections at the progenitor stage and somatosensory corticopontine topographic organization at postmitotic developmental stages. The study is very well written and the Figures are of high quality. The justification for many experimental steps could be clarified, and some are not clear until the discussion. The early Figures, although nice, seems somewhat tangential and equally the authors could instead add an investigation of the Emx1-cKO mice using anterograde tracer injections as in Figure 7 to assess the contribution of S1 directly to the increased corticopontine labelling. The dynamics of the KO model in combination with the utilization of the Thy1-YFP mouse line were not clear to me and could be improved to increase accessibility to a less specialized audience.

Major comments:

1. Why do the authors investigate only somatosensory and motor regions - especially given the high expression of Nr2fl in the caudolateral cortical regions? For instance, a comparison could have been made to the visual cortex from the start.
2. My instinct is that Figure 1 could be supplementary (if needed at all). It may be nice to have in case someone wants to consult the specific methodology after reading the main results but is not extensively referred to in the text and is overly complicated for a quick understanding of the experimental design. The flow and justification should be made clearer in the texts as one goes. Also, it is indicated in the flow chart that the Allen brain atlas data is only used for Figure 2 - however, Figure 3 figure legend also claims to be data from Allen. I assume this Figure labels in this schematic are wrong.
3. Similarly, while figure 2 is quite a nice figure, the significance of this comparison is more of a methodological control rather than a result. Figure 3 is also a nice confirmation of corticopontine

© 2022. Published by The Company of Biologists under the terms of the Creative Commons Attribution License (https://creativecommons.org/licenses/by/4.0/).

projections between mice and rats; although this is slightly more comprehensive and direct than what has been previously shown for S1 and M1/M2 (in studies cited by the authors), the amount of text and Figure space dedicated to this point seems excessive as it is not the main point or novel finding of the study. Perhaps Figure 2 and 3 could be combined and the point succinctly made with respect to the Allan brain atlas data with a confirmation from the lab's injections sites as a supplement. It seems to me that the entire point can be well made with the text from lines 184-189 alone.
4. Lines 221-230: The justification for examining both lines (and embryonic ages) is not fully clear. The intro (lines 94-102) also does not indicate why these two lines (representing developmental stages E9.5 and E11.5) are hypothesized to be interesting to compare.
5. The strategy of using the Thy1 reporter mice means that you are only seeing effects of the KO in a sub-population of selected neurons. Clearly, this was desired to focus on the corticospinal neurons, but this is not adequately explained. This also relies on the specification of this population for this purpose. How much heterogeneity is in the expression of the line. Further, this distribution (Figure 4B,B') is counter to the expression levels of Nr2f1 (Figure 7a). Is this of significance and why did the authors choose this approach? Line 258: 'as assessed by the YFP signal expression': but what is the logic for Thy1-driven YFP to be altered by Nr2f1? Although this is mentioned briefly in lines 226-229, this could be made more direct.
6. It is also unclear why the expression of YFP shifts to be more highly expressed in caudal regions in the KO lines. The pattern of results if just M1/M2 and S1 are taken into account makes sense (e.g. as summarized in Figure 8), but what is the implication for increased YFP expression in caudal regions? Is the increase in pontine labelling from just increased YFP signal in caudal regions or a true trade-off between CST and CP pathways? I am missing a clear explanation of the role of Nr2f1 in the designation of corticopontine fibers. If low gradient of Nr2f1 results in more corticospinal projections (e.g. in motor cortex) in controls, then knocking out Nr2f1 in progenitor cells results in more corticopontine fibers? But realistically what is being measured is more Thy1-driven YFP expression caudally, which may just highlight cortical regions that already have more corticopontine projections (in relation to corticospinal). For instance, if one adds the Nr2f1 gradient information to Figure 8, It is not clear to me what the direct relationship is here.
7. Related to above, why was Figure 7 only done in Nex-cKO mice? It would be informative to see the direct effects of the area specific projections in the Emx1-cKO in this regard.
8. In addition, some examples of actual injections sites (even in a supplementary Figure) would be helpful here since the mice were injected at P5, how does this affect the injection site volume/spread etc compared to the Allan brain data.
9. The semi-quantitative assessment in Figure 4F is confusing. Why not put regions of interest around the various areas and get a quantitative measure of layer 5 intensity? Or ideally, use image analysis tools for cell detection. The images seem like they would be suitable for this. Especially if the authors wish to do statistics based on this data, a less subjective and more quantitative measure is certainly required and not difficult to do. Additionally, the current scale is described as going from 0-4, but the mean is sometimes >4. So, did the scale actually go to 5? I appreciate the amount of work that went into this especially with regard to Supplementary Figure 4, but the subjective nature of the method does not seem necessary.
10. Line 288: calling the results an 'impaired cortical distribution of YFP expressing layer V neurons' seems to link to function. Perhaps 'disordered' is a better word here.
11. Lines 308-312: in Figure 5G and H, the variance across animals is very different and while both show the same trends, the significance changes because of the increased error in G. What is the source of this difference in variance in the measures of rostral and caudal area? This makes the result here difficult to interpret.
12. What is the implication of the increased fasciculation in Figure 5? Lines 322-324: again, functionally speaking, 'controls' seems like a strong word. Can the authors clarify the causal relationship to the fasciculation?
13. Much of the useful justification that would make the results easier to follow is in the discussion. I would suggest trying to (as concisely as possible since the results are already quite long) move/summarize some of this justification in key transition periods for the results.
E.g. info from section line 530, also 552.

Minor Comments:

- Figure 3. Please outline V1 if you want to indicate it in the figure - since all other areas are outlined, this suggests the entire caudal cortex is V1.

© 2022. Published by The Company of Biologists under the terms of the Creative Commons Attribution License (https://creativecommons.org/licenses/by/4.0/).

- Figure 3. Please indicate in B/I caudal-rostral, medial-lateral, dorsal-ventral. This will help with easy identification in C-G/J-N.
- Lines 217-221: this is quite a long, complex sentence. Consider rewording.
- Line 221: might be useful to briefly specify here what control animals are - littermate controls from methods?
- Line 413-414: different font.
- Figure 7: in C, is the left schematic really dorsal (to the top)? According to the guide on the right schematic, would this not be more like rostral? In reality it is more like a R-D angle, but these labels make the 3D views even more difficult to visualize. Perhaps an accompanying schema, perhaps a condensed version of that in Figure 2, would serve useful in the other figures as well.
- What is meant in lines 750-752 that the Franklin and Paxinos atlas was used when sections were matched with the Allen brain atlas? Why was this necessary?
- Line 755: what is the P8 in reference to? I only found reference to P21 and adult. Was this for the in situ? This is later specified as P7 (line 768), but P5 in Figure 7 legend (line 468). Please clarify ages.
- Sometimes they are referred to as Emx-cKO instead of Emx1-cKO.
- Line 721: missing closing bracket

Significance

The study offers a significant advance in our knowledge of how a specific molecular determinant contributes to the specification and organization of corticofugal projections. The authors have also made much of the data available in supplementary Figures and have presented the findings comprehensively. The study represents one of only a handful of such investigations of corticopontine projections in mice, compares in detail to the topography already described in detail in rats, and will be a welcome resource for those studying corticopontine and corticospinal projections in mice. Although the study is very targeted in scope in this regard, the combination of molecular patterning across development and adult anatomical organization means the study will appeal to a wider audience. My expertise is in neuroanatomical and functional aspects of corticofugal projections, not in the molecular patterning across development. In this regard, I have noted a number of areas in which it was unclear to me either the justification for the experimental design or the direct implication of the results. I hope these comments will allow the authors to broaden their target audience even further.

Referee Cross-commenting

I also agree with the comments from the other reviewers and echo the major concerns of reviewer 2 in this regard. I will also say that my assessment of 1-3 months for revisions was an optimistic underestimate. I agree that up to 6 months would be more appropriate as the other reviewers suggested.

Reviewer 2

Evidence, reproducibility and clarity

This manuscript from Chiara Tocco, Martin Ovsthus, and colleagues explores the consequences of inactivating the gene Nr2f1/COUP-TFI in cortical progenitors or post-mitotic neurons on the topography of the cortico-pontine projection. The group has extensive knowledge of Nr2f1 and its effects on cortical mapping and laminar development and the consequences of its inactivation on the development of sensory-motor systems. Based on their previous work, the rationale for looking into the role of Nr2f1 on the development of cortico-pontine axons is strong and should yield important information on underexplored aspects of its biology.

For this, the authors use 3 complementary approaches: analysis of publicly available tracing data from the Allen Brain Institute, histological analysis of labeled neurons in the Thy1.1-YFP H line crossed with Emx1 and Nex cre lines, and viral tracing in pups in Nex cre lines. Based on these experiments and with the help of alignment and standardization tools for 2d histology, the authors conclude that the post-mitotic absence of Nr2f1 affects the topography of cortico-pontine projections.

© 2022. Published by The Company of Biologists under the terms of the Creative Commons Attribution License (https://creativecommons.org/licenses/by/4.0/).

The two most convincing results presented here are 1) The concentric organization of cortico-pontine projections from the Allen Institute dataset analysis 2) The presence of fasciculation defects following the Emx1 and Nex-cre deletions of Nr2f1.

Otherwise, the methodology used throughout the rest of the manuscript and the presented results do not strongly support the conclusions of this work, as shown in Figure 8.

Major points:

An important issue is the use of the Thy1.1 H-line reporter to label layer V neurons in the cortex instead of relying more strongly on viral tracings done with stereotaxic injections. The changes in GFP expression patterns both in the cortex or in the Pons in this line crossed with the mutant lines are difficult to interpret because:

- The expression of YFP in the cortex is clearly modified in the mutant. Still, it is unclear whether this is due to the absence of layer V neurons, their respecification to other layer types, or merely to differential regulation of the transgene expression in the mutant lines. To answer these questions, the authors could have combined labeling of the YFP with laminar cortical markers and estimate the signal at the single-cell level on sections, to estimate the molecular identity of Thy1.1+ neurons in the mutants. Without precise information on the molecular nature of the labeled Thy1.1YFP neurons in the mutant cortex, it is impossible to interpret potential changes in the distribution of YFP+ fibers in the pons.
- The density of the labeling in the Pons is extremely high, and the phenotypes showed in figure 6 are not clear. It is appreciated that the authors included a complete survey of the cases in the supplementary information, but this highlights the strong variability in the precise signal distribution across cases and the phenotypes are not convincing. An unbiased quantification with statistics of the signal distribution could have helped. Because fibers from the whole cortex are labeled, this experiment doesn't allow to conclude on the effect of the mutation on the pontine projections, apart from the fact that they are disorganized in both mutants (Fig. 5).
- Likewise, it is not easy to interpret differences in the size of the labeled CST tract, as it could be due to either guidance defects upstream, loss of layer V neurons, loss of YFP expression in the mutants, or all of the above, so the experiment shown in Fig 5 is potentially interesting, but not informative on its own and should be combined with a complete survey of the tract in the rest of the brain, and complemented with tracing methods.

(maybe a typographic error, but mouse number 18166 appears twice as both a mutant and control in the supplementary information).

There are also issues with the viral tracing experiments:

- The drawn maps for the injection assume a wild-type map, which is not the case in the mutants for area sizes. Therefore the injections in the mutant brains may not be in the actual region shown. Also, the map doesn't include multi-sensory integrative regions, which have an extremely different connectivity pattern than sensory-motor regions. As a result, the sizes of V1, S1, and A1 are too large, as these integrative regions occupy territories in between, and some injection points may fall in one of these regions.
- The size of the injection spot shown may be misleading: considering the considerable amount of virus injected in the first post-natal week (500nL to 1µL at 10^12!), it is likely that the labeled neurons are located in a much larger area than shown on the figure. The authors should provide the actual histological data in the injection sites to support the figure and the precision of the injection point. Moreover, raw images of the pontine projections would be welcome on top of the segmented images shown.
- It is difficult to parse the mutant phenotype from the results of the viral tracings: control injections in the somatosensory map show widely different projection patterns: panels G, H, and I have close injection sites in controls, but very different projection patterns. Could this be due to registration errors? Also, figure 3 suggests that the mediolateral location of the neuron is an important factor in determining pontine topography. Maybe a color map as in Figure 3H-N would be more informative to compare mutants and controls. However, in the current state, the variability of injection results in controls and mutants alike would prevent the reader from drawing strong conclusions on the phenotype.

© 2022. Published by The Company of Biologists under the terms of the Creative Commons Attribution License (https://creativecommons.org/licenses/by/4.0/).

- Finally, as the claim is that Emx1cre based deletion of Nr2f1 has a different phenotype, injections in this background would be mandatory to draw this conclusion.

The results presented in Figures 3 and 7 don't strongly support the summary of figure 8. Figure 3 shows that the mediolateral location of the neuron is the main factor that drives the topography of the Pons, as sensory and motor projections (likely originating from a similar lateral position) overlap in the caudal pons, a fact not represented in the summary on Figure 8. Moreover, since viral injections have not been done in the Emx1 mutant, the authors cannot conclude on the organization of these projections based solely on the results of figure 6, which are not precise enough to assess a normal topography.

To obtain a better description of the phenotype of both mutants, the authors should have relied on viral mapping done in adult animals, where it is possible to carry precise stereotaxic injections of smaller volumes (10-50nL) of virus in the cortex. It would have been possible also to use dual-injections strategies to label cortico-pontine projecting neurons specifically. Unfortunately, the methodology presented here only gives partial and hard to interpret data.

Minor points:

- Figure 3: the medio, lateral and anterior axis should be shown in panels B-G and I-N
- Figure 4: fully quantitative count of the neurons should be done, instead of the qualitative (not "semi-quantitative"!) scale used here.
- Figure 5: can you indicate the standard deviation in the curves E, F?
- Methods: why is affine transformation done and not deforming transformation? This may lead to a more accurate alignment.

Significance

This study carries the work done by the group on the importance of post-mitotic expression of areal-specification genes. While a lot has been done on the role of COUP-TFI on cortical development, it is important to assess the consequences mapping errors can have on the topography of projections and how genes controlling the nature of cortical areas can also act directly on axon guidance. While this has been done in other contexts, the focus on the post-mitotic role of these genes here is noteworthy and interesting.

Referee Cross-commenting

I agree with the comments from other reviewers, some of which confirm also my major concerns that 1) More precise analysis with viral tracings are warranted and 2) The use of the Thy1.1-YFP reporter in a mutant context makes it difficult to interpret the results.

Reviewer 3

Evidence, reproducibility and clarity

In this manuscript, Tocco et al investigate a possible role of Nr2f1 expressed in cerebral cortical neurons in regulating the topography of corticopontine projections. The authors employ two different conditional deletions of Nr2f1 to delete the gene in precursors or postmitotic neurons. By crossing these animals with a Thy-YFPO reporter line they can quantify the number of corticospinal L5 neurons and describe the topography of their projections in the pontine nucleus. This is a beautiful approach to investigate if intrinsic information in cortical progenitors and postmitotic neurons determines subcortical connectivity.

The first set of experiments show that Nrf21 determines area-specific distributions and total numbers of L5 corticospinal neurons. In general, the expression of YFP show decreases in rostral areas and increases in caudal regions. When Nr2f1 is deleted in postmitotic neurons the net result seems an overall reduction of the total number of YFP expressing neurons. Deletions in precursors cells change the distribution, and not so much the numbers of YFP neurons in the cortical areas. The authors conclude that Nrf2 plays different roles in the differentiation of L5 precursors and

© 2022. Published by The Company of Biologists under the terms of the Creative Commons Attribution License (https://creativecommons.org/licenses/by/4.0/).

postmitotic neurons. Quantifications of these experiments are adequate and conclusions are correct although not surprising taking into account previous work from the group.

In the second part of the study, the authors analyze the topography of corticospinal and corticopontine projections in mutants in which Nrf21 is genetically deleted to investigate if the different cortical distribution and presumably a modified identity, correlate with distinct mapping. They perform viral injections and beautiful image reconstructions. This is an elegant approach. The main obstacle here is that the descriptions of the distribution of projections is qualitative and not quantitative. The number of injections is also restricted to two or three injections per area. With those numbers and without quantifications the study does not allow to describe the variability of the experimental method and more importantly, the intrinsic variability of projections within each of the cortical areas tested. This is an important obstacle for any strong conclusions. In WT animals, for example, the results suggest rather noisy maps.

Major concerns:

The lack of quantifications and the limited number of injections make the study more descriptive than conclusive.

The title somehow contradicts the experimental results, which are mainly obtained from mutant precursors. The abstract needs revision as it does not completely accurately reflect the conclusions that can be obtained from the results.

Minor concerns:

In Fig 6, panels E,I and M, the drawings appear to represent elongated images of the corticopontine sections in B-L .

Significance

The brain is a complex network of connections arranged with astounding selectivity and topographic precision but we still do not understand how they are built and to what extent they are driven by intrinsic and extrinsic information. This work aims to understand if Nr2f2 intrinsically determines the connectivity and topography of projections from corticospinal neurons.
Audience: researches in neurodevelopment, neurodevelopmental disorders, modeling of brain networks, neuronal differentiation, and diseases of the nervous system.
Expertise: cortical development

Referee Cross-commenting

Dear all, I also agree with your reviews and comments.

<u>Author response to reviewers' comments</u>

Reviewer #1 (Evidence, reproducibility and clarity (Required)):

Tocco et al examine the changes in the topographic organization of corticopontine projections and infer the ratio of corticopontine versus corticospinal projections from motor and somatosensory cortex after knocking out the expression of Nr2f1 at two developmental timepoints. They conclude that Nr2f1 acts as a molecular determinant of the ratio between corticopontine and corticospinal projections at the progenitor stage and somatosensory corticopontine topographic organization at postmitotic developmental stages. The study is very well written and the Figures are of high quality. The justification for many experimental steps could be clarified, and some are not clear until the discussion. The early Figures, although nice, seems somewhat tangential and equally the authors could instead add an investigation of the Emx1-cKO mice using anterograde tracer injections as in Figure 7 to assess the contribution of S1 directly to the increased corticopontine labelling. The dynamics of the KO model in combination with the utilization of the

© 2022. Published by The Company of Biologists under the terms of the Creative Commons Attribution License (https://creativecommons.org/licenses/by/4.0/).

Thy1-YFP mouse line were not clear to me and could be improved to increase accessibility to a less specialized audience.

→***Authors response:*** We thank the reviewer for considering our study well-written with high quality figures. As recommended, we have made substantial efforts to clarify the issues raised, and justified why we did not include the tracing part of the *Emx1-cKO* mouse model. Our point-by-point responses are given below.

Major comments:

1. Why do the authors investigate only somatosensory and motor regions - especially given the high expression of Nr2fl in the caudolateral cortical regions? For instance, a comparison could have been made to the visual cortex from the start.

→***Authors response:*** Thank you for the interesting and relevant question. We were primarily interested in the topography of corticopontine projections related to cerebellar functions with tactile representations and the majority of those originate from the somatosensory (S1) cortex. The somatosensory projections of the cerebro-cerebellar system constitute the largest proportion of the system, with highly organized projections to central parts of the pontine nuclei (Leergaard et al., 2000a; Leergaard et al., 2000b), while projections from the visual cortex are located in the lateral and rostral periphery of the pontine nuclei, and therefore not very amenable for distinguishing changes in topography. In addition, our previous work on Nr2f1 showed dramatic changes in the S1 area which takes a motor-like identity upon loss of Nr2f1 (Alfano et al., 2014b; Armentano et al., 2007b). So, while it would be interesting to assess impact of Nr2f1 on topographical organization of several subcortical projection systems, we deemed more logical to first investigate impact on the topographic organization in somatosensory projections in comparison with projections from the motor cortex. We have added a specification of this important point in the Introduction (page 3).

2. My instinct is that Figure 1 could be supplementary (if needed at all). It may be nice to have in case someone wants to consult the specific methodology after reading the main results but is not extensively referred to in the text and is overly complicated for a quick understanding of the experimental design. The flow and justification should be made clearer in the texts as one goes. Also, it is indicated in the flow chart that the Allen brain atlas data is only used for Figure 2 - however, Figure 3 figure legend also claims to be data from Allen. I assume this Figure labels in this schematic are wrong.

→***Authors response:*** We thank the reviewer for pointing out the mistake in Figure 1. We have now simplified the flowchart of the different processing and analytic steps and adapted it to some of the figures in relation to the type of experimental approach. We consider that it is important to keep this corrected workflow as Figure 1, so that the reader can easily go back to it. In this way single procedures can be referred in the text, and the workflows should be easier to follow when reading the main text and figures.

3. Similarly, while figure 2 is quite a nice figure, the significance of this comparison is more of a methodological control rather than a result.

→***Authors response:*** The purpose of this figure was twofold. On one side, we wished to present the published data from the *Allen Mouse Brain Connectivity Atlas* used as supplementary controls for one of our tract tracing paradigms; on the other side, we aimed to demonstrate that the 3D point data derived from tract tracing experiments in wild-type mice from the Allen institute, and our control animals yielded comparable results. We have therefore followed the reviewer's advice and combined Figures 2 and 3 into a new Figure 2, which exclusively presents the 3D visualization of topographical organization based on tract tracing data from the *Allen Mouse Brain Connectivity Atlas* (see also below).

Figure 3 is also a nice confirmation of corticopontine projections between mice and rats; although this is slightly more comprehensive and direct than what has been previously shown for S1 and M1/M2 (in studies cited by the authors), the amount of text and Figure space dedicated to this point seems excessive as it is not the main point or novel finding of the study. Perhaps Figure 2 and 3 could be combined and the point succinctly made with respect to the Allan brain atlas data with a confirmation from the lab's injections sites as a supplement. It seems to me that the entire

© 2022. Published by The Company of Biologists under the terms of the Creative Commons Attribution License (https://creativecommons.org/licenses/by/4.0/).

point can be well made with the text from lines 184-189 alone.

→***Authors response:*** We agree that the demonstration of topographical organization in the mouse pontine nuclei is a secondary point of the study, but we consider it very important to include, since our demonstration that the topographical distribution patterns in mice resembles those demonstrated earlier in rats is a novel finding, and essential to the interpretation of our tract tracing results in *Nex-cKO* mice. We have therefore followed the Reviewer's advice and combined Figures 2 and 3 (now Figure 2), with downscaled panels and reduced the descriptions in the main text substantially.

4. Lines 221-230: The justification for examining both lines (and embryonic ages) is not fully clear. The intro (lines 94-102) also does not indicate why these two lines (representing developmental stages E9.5 and E11.5) are hypothesized to be interesting to compare.

→***Authors response:*** We apologize for this confusion, and have added a few words at pages 5 and 9 in the Result section and page 22 in the Discussion part for clarifying the reason of using two independent conditional lines. In brief, the *Emx1-cKO* line challenges the role of Nr2f1 in cortical progenitors and postmitotic progeny (thus neurons) by acting early during development (at E9.5), while the *Nex-cKO* line challenges Nr2f1 function exclusively in postmitotic neurons (at E11.5), since normal expression is maintained in progenitors. This specific difference helps us distinguishing whether Nr2f1 expression acts at the level of progenitor cells or differentiating neurons during establishment of corticopontine topography.

5. The strategy of using the Thy1 reporter mice means that you are only seeing effects of the KO in a sub-population of selected neurons. Clearly, this was desired to focus on the corticospinal neurons, but this is not adequately explained. This also relies on the specification of this population for this purpose. How much heterogeneity is in the expression of the line. Further, this distribution (Figure 4B,B') is counter to the expression levels of Nr2f1 (Figure 7a). Is this of significance and why did the authors choose this approach? Line 258: 'as assessed by the YFP signal expression': but what is the logic for Thy1-driven YFP to be altered by Nr2f1? Although this is mentioned briefly in lines 226-229, this could be made more direct.

→***Authors response:*** It is correct that our aim was to use a reporter line expressed only in subcerebral projection neurons of layer V. The Thy1-eYFP line is perfectly suitable for this approach and is reported as a reliable and reproducible layer V reporter line in several studies. There is no particular heterogeneity reported in this line, although expression levels can slightly change between animals. Expression differences may also be ascribed to minor variations in the experimental fixation procedures. The YFP expression follows the physiological distribution of subcortical layer V projections, with a high density in motor cortex and a gradually decreased distribution in S1 and more caudal areas (Porrero et al., 2010). Since the distribution of layer V neurons changes in the absence of Nr2f1, as previously reported in our studies (Alfano et al., 2014c; Armentano et al., 2007a; Tomassy et al., 2010), the distribution of Thy1-YFP signal will consequently be altered as a read-out of layer V changes. We thus asked whether these alterations in layer V distribution observed in the two mutant lines would be also reflected in layer V axons projecting towards the pons. It turned out that this is the case. For example, high density of layer V neurons in S1 (indicated as a higher YFP signal) is translated in an abnormally higher corticopontine innervation. We realize that our paradigm was insufficiently explained, and to clarify this we have revised the Result section (page 10).

6. It is also unclear why the expression of YFP shifts to be more highly expressed in caudal regions in the KO lines. The pattern of results if just M1/M2 and S1 are taken into account makes sense (e.g. as summarized in Figure 8), but what is the implication for increased YFP expression in caudal regions? Is the increase in pontine labelling from just increased YFP signal in caudal regions or a true trade-off between CST and CP pathways? I am missing a clear explanation of the role of Nr2f1 in the designation of corticopontine fibers. If low gradient of Nr2f1 results in more corticospinal projections (e.g. in motor cortex) in controls, then knocking out Nr2f1 in progenitor cells results in more corticopontine fibers? But realistically what is being measured is more Thy1-driven YFP expression caudally, which may just highlight cortical regions that already have more corticopontine projections (in relation to corticospinal). For instance, if one adds the Nr2f1 gradient information to Figure 8, It is not clear to me what the direct relationship is here.

→***Authors response:*** We fully appreciate the reviewer question, and apologize for insufficient explanation of our experimental paradigm. We used the Thy1-H-eYFP line as means to label

© 2022. Published by The Company of Biologists under the terms of the Creative Commons Attribution License (https://creativecommons.org/licenses/by/4.0/).

exclusively layer V projection neurons (corticospinal and corticopontine) and investigate the corticopontine axonal projection topography. We expected that an abnormal cortical distribution of YFP-expressing layer V neurons would be translated into altered corticospinal projections and/or corticopontine topographic mapping. This is the reason why we first assessed the spatial organization of YFP-expressing layer V cortical distribution in mutant adults compared to control animals. Since the YFP reporter line labels layers V projecting neurons across the entire cerebral cortex, high caudal expression of YFP (particularly in the visual cortex) implies a higher density of layer V neurons in this area (mainly corticotectal projection neurons). We have mentioned this phenomenon in our recent paper (Bertacchi et al., 2020), showing that in the absence of Nr2f1, progenitors in the caudal cortex tend to proliferate more and to produce a higher number of deep layer neurons. Since the present study focuses on motor and somatosensory cortices for the reasons mentioned above, we have not followed the trajectory of this excess of layer V projections neurons in the visual areas. This might be very interesting with respect to retino-geniculate topography, which is not the topic of this study. Finally, the gradient of Nr2f1 plays distinct roles along the cortex, as described in our recent review (Bertacchi et al., 2019). This implies that in its absence, S1 identity (where Nr2f1 is expressed) is not properly specified and motor identity (where Nr2f1 is not expressed) takes over since not repressed by Nr2f1 in S1. In addition, overexpression of Nr2f1 in motor cortex leads to a change in sensory identity, as shown by markers and thalamocortical projections (Alfano et al., 2014a). In brief, Nr2f1 is necessary and sufficient to impart sensory identity in the developing neocortex. To better explain our paradigm, we have revised the Introduction (page 5), where we also point to our earlier findings.

7. Related to above, why was Figure 7 only done in Nex-cKO mice? It would be informative to see the direct effects of the area specific projections in the Emx1-cKO in this regard.

→***Authors response:*** This is a legitimate question, and we apologize the reviewer for not having clarified the choice of the *Nex-cKO* model. The main reasons are now explained at the beginning of the paragraph dealing with the viral injection experiments. Since we found unexpected aberrant and homogenous corticopontine projections in the *Emx1-cKO* line, we rationalized that tracing the cortical origin of all the projections would not give us a clear answer on topographic shifts, by making the study of topographic mapping very difficult to interpret. We thus decided to focus on the *Nex-cKO* mice, which topographical shifts was already better defined with the help of the reporter Thy1-YFP, and therefore more suitable for experimental area-specific tract tracing studies.

8. In addition, some examples of actual injections sites (even in a supplementary Figure) would be helpful here since the mice were injected at P5, how does this affect the injection site volume/spread etc compared to the Allan brain data.

→***Authors response:*** We fully agree with the reviewer that transparency and full documentation tracer injection sites are important and will therefore also share all microscopic image data. But to improve the documentation of our experimental material, we have added microscopic images documenting representative injection sites and pontine labelling to new Figures 6 and 7 (previously Figure 7) and will create a new supplementary figure (in the following revised version) showing the centres of all injection sites, together with representative documentation of the pontine labelling. At P5, the motor and somatosensory cortical areas are well defined and amenable to inject at the expected locations.

9. The semi-quantitative assessment in Figure 4F is confusing. Why not put regions of interest around the various areas and get a quantitative measure of layer 5 intensity? Or ideally, use image analysis tools for cell detection. The images seem like they would be suitable for this. Especially if the authors wish to do statistics based on this data, a less subjective and more quantitative measure is certainly required and not difficult to do. Additionally, the current scale is described as going from 0-4, but the mean is sometimes >4. So, did the scale actually go to 5? I appreciate the amount of work that went into this especially with regard to Supplementary Figure 4, but the subjective nature of the method does not seem necessary.

→***Authors response:*** We thank the Reviewer for pointing out the inconsistency of the numbers in Figure 4F. This is the result of how the analysis was conducted. For instance, every image was aligned with the corresponding Allen brain atlas plate, for area segmentation. Then, an intensity score was attributed for each area. Admittedly, in a subset of images, the YFP signal was not only observed in layer V, but also in layer VI, possibly due to an artefact of alignment between our

© 2022. Published by The Company of Biologists under the terms of the Creative Commons Attribution License (https://creativecommons.org/licenses/by/4.0/).

experimental images and the atlas plates. Knowing that the YFP reporter gene is specifically expressed in layer V, we decided to independently score both layers indicated by the atlas, and then sum up the two values, before calculating the average value among different sections. We understand now that this could lead to imprecise quantification, and we propose a new automatic Image J macro, which will allow us to count spots by area of interest in the brain. The spots are considered as specific staining using a threshold based on intensity and shape of the elements and the composite RGB segmentation atlas as a mask for the region of interest. This will result in a more reliable analysis of the distribution of YFP positive cells among the different functional areas. *We have now added a detailed pipeline with some examples as an ANNEX at the end of this document* (pages 16,17).

10. Line 288: calling the results an 'impaired cortical distribution of YFP expressing layer V neurons' seems to link to function. Perhaps 'disordered' is a better word here.

→***<u>Authors response:</u>*** Fully agreed. We have changed it as suggested in our revised manuscript.

11. Lines 308-312: in Figure 5G and H, the variance across animals is very different and while both show the same trends, the significance changes because of the increased error in G. What is the source of this difference in variance in the measures of rostral and caudal area? This makes the result here difficult to interpret.

→***<u>Authors response:</u>*** We agree that this is the case, and that the reasons might come from the more severe phenotype of the *Emx1-cKO* tract that renders the quantification more difficult to assess. However, despite the variance observed, we see a clear trend, and we have now acknowledged this observation in the text at page 12. However, we do not believe that it impacts our overall conclusion.

12. What is the implication of the increased fasciculation in Figure 5? Lines 322-324: again, functionally speaking, 'controls' seems like a strong word. Can the authors clarify the causal relationship to the fasciculation?

→***<u>Authors response:</u>*** We agree with the reviewer that we have no direct proof of a causal relationship of Nr2f1 to axonal fasciculation. We have therefore change the sentence to: "…these data show that loss of Nr2f1 expression (both in cortical progenitor cells and postmitotic cells) affects the diameter, shape and degree of fasciculation of the CST originating from layer V neurons.".

13. Much of the useful justification that would make the results easier to follow is in the discussion. I would suggest trying to (as concisely as possible since the results are already quite long) move/summarize some of this justification in key transition periods for the results.
E.g. info from section line 530, also 552.

→***<u>Authors response:</u>*** We agree that our Results section could benefit from some additional information, and have therefore added explanatory text in the Result section, as suggested by the Reviewer's suggestion.

<u>Minor Comments:</u>

- Figure 3. Please outline V1 if you want to indicate it in the figure - since all other areas are outlined, this suggests the entire caudal cortex is V1.

→***<u>Authors response:</u>*** We thank the Reviewer for pointing this out and have removed annotations for areas not relevant for the present investigations.

- Figure 3. Please indicate in B/I caudal-rostral, medial-lateral, dorsal-ventral. This will help with easy identification in C-G/J-N.

→***<u>Authors response:</u>*** This is now Figure 2, to which we have added a new top row which includes 3D renderings of the pontine nuclei, in view from sagittal and ventral, that aid understanding the angle of view of the dot maps shown below. We have added explanation to the Figure legend, and removed the diagonal cross indicating dorsal and ventral relative to the long axis of the brain stem at level of the pons (see also the Reviewers comment to Figure 7, below).

© 2022. Published by The Company of Biologists under the terms of the Creative Commons Attribution License (https://creativecommons.org/licenses/by/4.0/).

- Lines 217-221: this is quite a long, complex sentence. Consider rewording.

→***Authors response:*** We agree, and have rephrased using 2 separate sentences.

- Line 221: might be useful to briefly specify here what control animals are - littermate controls from methods?

→***Authors response:*** We have now specified that controls are indeed littermate controls.

- Line 413-414: different font.

→***Authors response:*** This has been corrected

- Figure 7: in C, is the left schematic really dorsal (to the top)? According to the guide on the right schematic, would this not be more like rostral? In reality it is more like a R-D angle, but these labels make the 3D views even more difficult to visualize. Perhaps an accompanying schema, perhaps a condensed version of that in Figure 2, would serve useful in the other figures as well.

→***Authors response:*** The oblique orientation of the brain stem at level of the pons can indeed be confusing. We have therefore edited the orientation annotations and added small images of the Allen Mouse Brain Atlas to make the orientation more clear.

- What is meant in lines 750-752 that the Franklin and Paxinos atlas was used when sections were matched with the Allen brain atlas? Why was this necessary?

→***Authors response:*** This is an error related to an early stage preliminary analysis, which became obsolete after all images were spatially registered to the Allen mouse brain atlas. The sentence has been deleted.

- Line 755: what is the P8 in reference to? I only found reference to P21 and adult. Was this for the in situ? This is later specified as P7 (line 768), but P5 in Figure 7 legend (line 468). Please clarify ages.

→***Authors response:*** We apologize for the confusion, and have now corrected the ages.

- Sometimes they are referred to as Emx-cKO instead of Emx1-cKO.
- Line 721: missing closing bracket.

→***Authors response:*** We have now uniformed *Emx-cKO* to *Emx1-cKO*.

Reviewer #1 (Significance (Required)):

The study offers a significant advance in our knowledge of how a specific molecular determinant contributes to the specification and organization of corticofugal projections. The authors have also made much of the data available in supplementary Figures and have presented the findings comprehensively. The study represents one of only a handful of such investigations of corticopontine projections in mice, compares in detail to the topography already described in detail in rats, and will be a welcome resource for those studying corticopontine and corticospinal projections in mice. Although the study is very targeted in scope in this regard, the combination of molecular patterning across development and adult anatomical organization means the study will appeal to a wider audience. My expertise is in neuroanatomical and functional aspects of corticofugal projections, not in the molecular patterning across development. In this regard, I have noted a number of areas in which it was unclear to me either the justification for the experimental design or the direct implication of the results. I hope these comments will allow the authors to broaden their target audience even further.

→***Authors response:*** We thank the reviewer for appreciating the novelty of this study and the importance and advance in the field of topographic control of corticofugal projections.

Referee Cross-commenting

I also agree with the comments from the other reviewers and echo the major concerns of reviewer

© 2022. Published by The Company of Biologists under the terms of the Creative Commons Attribution License (https://creativecommons.org/licenses/by/4.0/).

2 in this regard. I will also say that my assessment of 1-3 months for revisions was an optimistic underestimate. I agree that up to 6 months would be more appropriate as the other reviewers suggested.

Reviewer #2 (Evidence, reproducibility and clarity (Required)):

This manuscript from Chiara Tocco, Martin Ovsthus, and colleagues explores the consequences of inactivating the gene Nr2f1/COUP-TFI in cortical progenitors or post-mitotic neurons on the topography of the cortico-pontine projection. The group has extensive knowledge of Nr2f1 and its effects on cortical mapping and laminar development and the consequences of its inactivation on the development of sensory-motor systems. Based on their previous work, the rationale for looking into the role of Nr2f1 on the development of cortico-pontine axons is strong and should yield important information on underexplored aspects of its biology. For this, the authors use 3 complementary approaches: analysis of publicly available tracing data from the Allen Brain Institute, histological analysis of labeled neurons in the Thy1.1-YFP H line crossed with Emx1 and Nex cre lines, and viral tracing in pups in Nex cre lines. Based on these experiments and with the help of alignment and standardization tools for 2d histology, the authors conclude that the post-mitotic absence of Nr2f1 affects the topography of cortico-pontine projections. The two most convincing results presented here are 1) The concentric organization of cortico-pontine projections from the Allen Institute dataset analysis 2) The presence of fasciculation defects following the Emx1 and Nex-cre deletions of Nr2f1.

Otherwise, the methodology used throughout the rest of the manuscript and the presented results do not strongly support the conclusions of this work, as shown in Figure 8.

→***Authors response:*** We remain confident that our findings of altered organization of corticopontine projections in *Emx1-cKO* and *Nex-cKO* brains are robust, but understand that the Reviewer may found discrepancies between our data and summary Figure 8. We have therefore substantially revised the summary panels and placed them in the relevant main figures, the new Figures 5 and 7. A new model has been now added in Figure 8. This summarized our findings with the ones in the literature. We have now clarified the issues pointed out by the reviewer, and adapted the text accordingly, and similarly to Reviewer 1, we have justified why we did not include the tracing part of the *Emx1-cKO* mouse model. Our point-by-point responses are given below.

Major points:

1. An important issue is the use of the Thy1.1 H-line reporter to label layer V neurons in the cortex instead of relying more strongly on viral tracings done with stereotaxic injections. The changes in GFP expression patterns both in the cortex or in the Pons in this line crossed with the mutant lines are difficult to interpret because: - The expression of YFP in the cortex is clearly modified in the mutant. Still, it is unclear whether this is due to the absence of layer V neurons, their respecification to other layer types, or merely to differential regulation of the transgene expression in the mutant lines. To answer these questions, the authors could have combined labeling of the YFP with laminar cortical markers and estimate the signal at the single-cell level on sections, to estimate the molecular identity of Thy1.1+ neurons in the mutants. Without precise information on the molecular nature of the labeled Thy1.1YFP neurons in the mutant cortex, it is impossible to interpret potential changes in the distribution of YFP+ fibers in the pons.

→***Authors response:*** Our major aim was to use a reporter line expressed only in layer V neurons and follow the subcortical projection pattern along the whole cortex before focusing on the somatosensory and motor cortices, known to massively contribute to the pontine nuclei innervation. We consider that the Thy1-H line is perfectly suitable for this approach and is reported as a reliable and reproducible layer V reporter line in several studies. There is no particular heterogeneity reported in the Thy1-H line, although expression levels can slightly change between animals. Expression differences may also be ascribed to minor variations in the experimental fixation procedures.
It is well reported that the YFP expression follows the physiological distribution of subcortical layer V projections, with a high density in motor cortex and a gradually decreased distribution in S1 and more caudal areas (Porrero et al., 2010). Since the distribution of layer V neurons changes in the

© 2022. Published by The Company of Biologists under the terms of the Creative Commons Attribution License (https://creativecommons.org/licenses/by/4.0/).

absence of Nr2f1, as previously reported in our studies (Alfano et al., 2014c; Armentano et al., 2007a; Tomassy et al., 2010), the distribution of the Thy1-YFP signal will consequently be altered as a read-out of layer V changes. We thus asked whether these alterations in layer V distribution observed in the two mutant lines would be also reflected in the organization of their projections toward the pontine nuclei. It turned out that this is the case. For example, high density of layer V neurons in S1 (indicated as a higher YFP signal) is translated into an abnormally higher and confused corticopontine innervation.

We acknowledge that combined labelling of the YFP with laminar cortical markers, would give us more information on the molecular identity of Thy1-YFP+ neurons in the mutants. But this is not really the focus of this study, which instead was in part answered in our previous reports (Alfano et al., 2014b; Harb et al., 2016; Tomassy et al., 2010). Here, we are mainly interested in understanding whether proper cortical area mapping is involved in corticopontine topography since very little is known on the role of molecular determinants controlling corticopontine connectivity. Therefore, we consider that using a mouse model in which cortical area mapping is perturbed very early during development (without affecting subcerebral structures and including the pontine nuclei) is perfectly suitable for validating our initial hypothesis of a spatio-temporal establishment of area-specific targeting of corticopontine neurons.

2. The density of the labeling in the Pons is extremely high, and the phenotypes showed in figure 6 are not clear. It is appreciated that the authors included a complete survey of the cases in the supplementary information, but this highlights the strong variability in the precise signal distribution across cases and the phenotypes are not convincing. An unbiased quantification with statistics of the signal distribution could have helped. Because fibers from the whole cortex are labeled, this experiment doesn't allow to conclude on the effect of the mutation on the pontine projections, apart from the fact that they are disorganized in both mutants (Fig. 5).

→*Authors response:* The density of fluorescence expression in the pontine nuclei shown in Figure 6 (now Figure 5) and extensively documented in Supplementary Figs. 1 and 2, is variable across cases in terms of signal intensity, but not in terms of innervation patterns. Our microscopic images and semiquantitative analysis therefore focussed on the spatial distribution of signal expression, rather than on quantification of the signals. But, the collections of spatially corresponding images shown in Supp. Figure 2 clearly show reduced signal expression in central parts of the pontine nuclei in all Nex-cKO cases, compared to the controls. We maintain that these findings give strong indications, which are further supported by the subsequent tract tracing data.

3. Likewise, it is not easy to interpret differences in the size of the 9abelled CST tract, as it could be due to either guidance defects upstream, loss of layer V neurons, loss of YFP expression in the mutants, or all of the above, so the experiment shown in Fig 5 is potentially interesting, but not informative on its own and should be combined with a complete survey of the tract in the rest of the brain, and complemented with tracing methods.

→*Authors response:* We appreciate the reviewer's comment, however Figure 5 (now Fig.4) shows that the diameter of the fascicle at the level of the cerebral peduncle is not changed among groups (also quantified in panel G), which excludes any important loss of neurons or YFP expression in the mutant groups. What this figure shows is a different contribution of axons towards either the pontine nuclei (increased in the *Emx1-cKO* group but maintained in the *Nex-cKO* one) and a fasciculation defect past the pontine nuclei in both groups. A complete survey of the tract is a very interesting suggestion but could not be part of this study, since out of focus.

4. maybe a typographic error, but mouse number 18166 appears twice as both a mutant and control in the supplementary information.

→*Authors response:* We acknowledge the mistake in the supplementary figure 1 and confirm that it was indeed a typographic error. As reported in supplementary table 2, animal #18166 is a control case, whereas the correct number of the third *Emx1-cKO* animal is in fact the #18046. The numbers have been corrected in the new version of the figure.

There are also issues with the **viral tracing experiments**:

4. The drawn maps for the injection assume a wild-type map, which is not the case in the mutants for area sizes. Therefore, the injections in the mutant brains may not be in the actual region shown. Also, the map doesn't include multi-sensory integrative regions, which have an

© 2022. Published by The Company of Biologists under the terms of the Creative Commons Attribution License (https://creativecommons.org/licenses/by/4.0/).

extremely different connectivity pattern than sensory-motor regions. As a result, the sizes of V1, S1, and A1 are too large, as these integrative regions occupy territories in between, and some injection points may fall in one of these regions.

→***<u>Authors response:</u>*** The serial histological sections were carefully registered to the 3D reference atlas using non-linear adjustments, based on multiple anatomical landmarks. The injection site locations were mapped onto the reference atlas using 3D spatial coordinates (and not area identity) to allow comparisons of spatial the locations of injection site centres. The cortical areal identities are certainly changed in the mutants, this is key to the experimental paradigm of our study. The spatial accuracy of our methodology is validated in Figure 2 (now Supp. Fig. 3), which shows similar spatial distributions of data points representing corticopontine labelling obtained by independent tract-tracing studies in wild- type (Allen institute data) and our control experiments. Interestingly, our tracer injections in control experiments yield similar results as those in wild-types, which also strongly resemble our earlier findings reported in rats (Leergaard and Bjaalie, 2007). The area designation of the atlas are only used to indicate assumed motor or somatosensory area identity, as only the spatial coordinates were used to identify spatially corresponding experiments for our comparisons. The annotation labels for A1 and V1 are removed.

5. The size of the injection spot shown may be misleading: considering the considerable amount of virus injected in the first post-natal week (500nL to 1µL at 10^12!), it is likely that the labeled neurons are located in a much larger area than shown on the figure. The authors should provide the actual histological data in the injection sites to support the figure and the precision of the injection point. Moreover, raw images of the pontine projections would be welcome on top of the segmented images shown.

→***<u>Authors response:</u>*** We fully agree that full documentation of tracer injection sites is essential. All suitable microscopic image data will be publicly shared but following the Reviewer's advice we have now also added microscopic images of representative injection sites and pontine labelling to the new Figures 6 and 7 (previously Fig. 7). These images demonstrate that the tracer injections are spatially delimited with a relatively small extent. In addition, we will prepare <u>a new Supplementary Figure showing microscopic images of tracer injection site centres and pontine labelling (in the following revised version).</u>

6. It is difficult to parse the mutant phenotype from the results of the viral tracings: control injections in the somatosensory map show widely different projection patterns: panels G, H, and I have close injection sites in controls, but very different projection patterns. Could this be due to registration errors? Also, figure 3 suggests that the mediolateral location of the neuron is an important factor in determining pontine topography. Maybe a color map as in Figure 3H-N would be more informative to compare mutants and controls. However, in the current state, the variability of injection results in controls and mutants alike would prevent the reader from drawing strong conclusions on the phenotype.

→***<u>Authors response:</u>*** The tract tracing experiments illustrated in previous Figure 7 (panels F- I, but not panel J) very clearly show that tracer injections in somatosensory areas in *Nex-cKO* brains give rise to substantially different projection patterns compared to spatially corresponding injections in control brains. These differences in spatial distribution are very distinct, in particular for tracer locations in medial parts of S1. Given our newly achieved understanding of corticopontine topography in the mouse brain (cf. our analysis of *Allen Mouse Brain Connectivity data*) and our findings that tracer injections in control animals yield the same labelling patterns as public data for wild-types, the differences observed in the mutants cannot be ascribed to registration errors. We realize that Figure 7 was very information rich, and have therefore split it in two new figures (now Fig. 6 and 7), where Figure 6 demonstrates how tracer injections in motor cortex of mutants give similar results as injections in controls/wild-types, while Figure 7 shows how projections from somatosensory regions in mutants are very different from controls.

7. Finally, as the claim is that Emx1 cre based deletion of Nr2f1 has a different phenotype, injections in this background would be mandatory to draw this conclusion. à →***<u>Authors response:</u>*** As mentioned above for Reviewer 1, we decided not to do the tracing tract experiments in the *Emx1-cKO* line, because our Thy1-YFP analyses (now Fig. 5) showed an unexpected aberrant and homogenous corticopontine projections in these mutants, differently from the *Nex-cKO* line in which the shifts were more clear-cut. We think that tracing the cortical origin of all the projections in these mutants will fail to give us a clear answer on topographic

© 2022. Published by The Company of Biologists under the terms of the Creative Commons Attribution License (https://creativecommons.org/licenses/by/4.0/).

shifts by making the study of topographic mapping very difficult to interpret. We thus decided to focus on the *Nex-cKO* mice, which topographical shifts was already better defined, and therefore more suitable for experimental area-specific tract tracing studies. It is however implicit that topographical shifts are embedded in the phenotype of the *Emx1-cKO* brains, but because of the abnormal increase of corticopontine innervation in theses mutants, this shift becomes very difficult to discern.

8. The results presented in Figures 3 and 7 don't strongly support the summary of figure 8. Figure 3 shows that the mediolateral location of the neuron is the main factor that drives the topography of the Pons, as sensory and motor projections (likely originating from a similar lateral position) overlap in the caudal pons, a fact not represented in the summary on Figure 8. Moreover, since viral injections have not been done in the Emx1 mutant, the authors cannot conclude on the organization of these projections based solely on the results of figure 6, which are not precise enough to assess a normal topography.

→***Authors response:*** We agree that our summary diagram in Figure 8 was not reflecting the results observed in *Emx1-cKO* mice, and have therefore made separate diagrams summarizing findings achieved with our different paradigms (Fig. 4 and 5, respectively). The revised diagrams now better capture the main findings observed in our material. We decided instead to add a final summary figure (Fig. 8) including also data from the literature and illustrating that both, the cortex and pontine nuclei, are involved in corticopontine topography mapping.

9. To obtain a better description of the phenotype of both mutants, the authors should have relied on viral mapping done in adult animals, where it is possible to carry precise stereotaxic injections of smaller volumes (10-50nL) of virus in the cortex. It would have been possible also to use dual-injections strategies to label cortico-pontine projecting neurons specifically. Unfortunately, the methodology presented here only gives partial and hard to interpret data.

→***Authors response:*** Topographically organized corticopontine projections are established early in rodents (Leergaard et al., 1995; Mihailoff et al., 1984; O'Leary and Terashima, 1988), and injections at post-natal stages are normally accepted (e.g., Gu et al., 2017). Juvenile mice at P21 will thus display the same topographical projections as in adults, if anything slightly more widespread than in adult animals. Finding altered projection topography in young animals only strengthens our conclusions. As commented above, our tracer injection sites are very delimited and relatively small, as shown in new Fig. 6 and 7, and as we now will document in our new Supplementary Figure, showing overview of all injection sites. We strongly believe that we will provide enough documentation showing that our tract tracing results in control animals give similar results as shown in the public data collection of the Allen Institute, and that the topography of corticopontine projections are altered in mutant animals receiving tracer injections in somatosensory areas, but not in animals receiving tracer injections in motor areas.

Minor points:

- Figure 3: the medio, lateral and anterior axis should be shown in panels B-G and I-N

→***Authors response:*** We have rearranged the Figure (now Fig. 2) and added annotations indicating orientation for all panels.

- Figure 4: fully quantitative count of the neurons should be done, instead of the qualitative (not "semi-quantitative"!) scale used here.

→***Authors response:*** We are preparing a quantitative image analysis for assessing density of YFP expressing neurons in the cerebral cortex.

- Figure 5: can you indicate the standard deviation in the curves E, F?

→***Authors response:*** We understand the concern of the reviewer, but we think that the addition of standard deviation curves to panels E and F might affect the overall readability of the graphs. However, the reader can consult the complete summary of statistical analysis performed in this work, reported in supplementary table 4.

- Methods: why is affine transformation done and not deforming transformation? This may lead to a more accurate alignment.

© 2022. Published by The Company of Biologists under the terms of the Creative Commons Attribution License (https://creativecommons.org/licenses/by/4.0/).

→***Authors response:*** The spatial registration of images was indeed optimized using a secondary non-linear registration step with the tool VisuAlign, as mentioned several times in the Methods text. However, in the main description of the spatial alignment procedure in the Methods section, this description was missing. We have now added the missing text (lines 678-681).

Reviewer #2 (Significance (Required)):

This study carries the work done by the group on the importance of post-mitotic expression of areal-specification genes. While a lot has been done on the role of COUP-TFI on cortical development, it is important to assess the consequences mapping errors can have on the topography of projections and how genes controlling the nature of cortical areas can also act directly on axon guidance. While this has been done in other contexts, the focus on the post-mitotic role of these genes here is noteworthy and interesting.

→***Authors response:*** We thank the reviewer for appreciating the importance of post-mitotic expression of areal-specification genes in topographic mapping during cortical development.

Referee Cross-commenting

I agree with the comments from other reviewers, some of which confirm also my major concerns that 1) More precise analysis with viral tracings are warranted and 2) The use of the Thy1.1-YFP reporter in a mutant context makes it difficult to interpret the results.

Reviewer #3 (Evidence, reproducibility and clarity (Required)):

In this manuscript, Tocco et al investigate a possible role of Nr2f1 expressed in cerebral cortical neurons in regulating the topography of corticopontine projections. The authors employ two different conditional deletions of Nr2f1 to delete the gene in precursors or postmitotic neurons. By crossing these animals with a Thy-YFPO reporter line they can quantify the number of corticospinal L5 neurons and describe the topography of their projections in the pontine nucleus. This is a beautiful approach to investigate if intrinsic information in cortical progenitors and postmitotic neurons determines subcortical connectivity.

→***Authors response:*** We thank the reviewer for considering our study a beautiful approach to understand whether and how intrinsic cortical information influences subcortical connectivity.

The first set of experiments show that Nrf21 determines area-specific distributions and total numbers of L5 corticospinal neurons. In general, the expression of YFP show decreases in rostral areas and increases in caudal regions. When Nr2f1 is deleted in postmitotic neurons the net result seems an overall reduction of the total number of YFP expressing neurons. Deletions in precursors cells change the distribution, and not so much the numbers of YFP neurons in the cortical areas. The authors conclude that Nrf2 plays different roles in the differentiation of L5 precursors and postmitotic neurons. Quantifications of these experiments are adequate and conclusions are correct although not surprising taking into account previous work from the group.

In the second part of the study, the authors analyze the topography of corticospinal and corticopontine projections in mutants in which Nrf21 is genetically deleted to investigate if the different cortical distribution and presumably a modified identity, correlate with distinct mapping. They perform viral injections and beautiful image reconstructions. This is an elegant approach. The main obstacle here is that the descriptions of the distribution of projections is qualitative and not quantitative. The number of injections is also restricted to two or three injections per area. With those numbers and without quantifications the study does not allow to describe the variability of the experimental method and more importantly, the intrinsic variability of projections within each of the cortical areas tested. This is an important obstacle for any strong conclusions. In WT animals, for example, the results suggest rather noisy maps.

→***Authors response:*** We thank the reviewer for evaluating our image reconstruction work as an elegant approach. Below, we have responded to the reviewer's concern regarding quantifications.

© 2022. Published by The Company of Biologists under the terms of the Creative Commons Attribution License (https://creativecommons.org/licenses/by/4.0/).

Major concerns:

1. The lack of quantifications and the limited number of injections make the study more descriptive than conclusive.

→***Authors response:*** The reviewer correctly observes that there are variations in the amount and spatial distributions across tract tracing experiments. This well-known variation is closely related to the location and extent of the tracer injection sites. The exact zone of tracer uptake varies with type of tracer and application method and is very difficult to assess quantitatively. The only way to conduct exact quantitative tract tracing is to trace individual neurons, which is demanding and mostly suited to investigate detailed axonal trajectories. Since the purpose of our study was to detect whether or not topographical distributions were grossly altered in mutant mice, the opted for system level tracing of projections from neuronal populations, which is difficult quantify. Our study involves data from 34 tract tracing experiments (13 mutants, 10 controls and 11 wild types). Following tracer injections in motor areas M1 or M2 of 7 mutants (3 shown in new Fig. 6) we observe similar tracer distribution patterns as in controls, while in 5 out of 6 tracer injections in the somatosensory cortex of mutants (all 6 shown in Fig. 7) yielded highly different labelling patterns compared to controls. We are convinced that these observations warrant the conclusion that topographical distributions are altered in somatosensory but not in motor projections of *Nex-cKO* mice.

2. The title somehow contradicts the experimental results, which are mainly obtained from mutant precursors. The abstract needs revision as it does not completely accurately reflect the conclusions that can be obtained from the results.

→***Authors response:*** We thank the Reviewer for this comment, but see that there may be a misunderstanding. The title clearly says that the defect in topographic mapping derives from lack of postmitotic (i.e., neuronal) Nr2f1 expression, and not of precursors. Indeed, while loss of Nr2f1 in precursor cells leads to an imbalanced ratio between corticopontine and corticospinal (with a higher rate towards the corticopontine population), loss of Nr2f1 in neuronal cells results in topographic shifts that we were able to pinpoint from the S1 subareas and not from motor areas. We think that the title and abstract appropriately reflects these conclusions.

Minor concerns:

In Fig 6, panels E,I and M, the drawings appear to represent elongated images of the corticopontine sections in B-L .

→***Authors response:*** The images shown in Figure 6E,I,N (now in Fig. 5) may appear elongated since they are shown in perspective, viewed obliquely from ventro-medial. Their spatial proportions have not been altered. It should also be noted that the dot maps only represent signal expression in the pontine nuclei, not signals in the longitudinal fibers of the pons. To better clarify the angle of view for the different panels, we have added several new orientation annotations.

Reviewer #3 (Significance (Required)):

The brain is a complex network of connections arranged with astounding selectivity and topographic precision but we still do not understand how they are built and to what extent they are driven by intrinsic and extrinsic information. This work aims to understand if Nr2f2 intrinsically determines the connectivity and topography of projections from corticospinal neurons.

Audience: researches in neurodevelopment, neurodevelopmental disorders, modeling of brain networks, neuronal differentiation, and diseases of the nervous system.

Expertise: cortical development

→***Authors response:*** We thank the reviewer for appreciating the importance of understanding intrinsic and extrinsic mechanisms in corticopontine topography and our contribution in this issue.

Referee Cross-commenting

Dear all, I also agree with your reviews and comments.

© 2022. Published by The Company of Biologists under the terms of the Creative Commons Attribution License (https://creativecommons.org/licenses/by/4.0/).

ANNEX: Pipeline for the automatic quantification of YFP-positive cortical neurons

We propose to perform this analysis by taking advantage of an ImageJ macro that allows automatic counting of spots by area of interest in the brain. The spots are considered as specific staining using a threshold based on intensity and shape of the elements and the composite RGB segmentation atlas as a mask for the regions of interest.

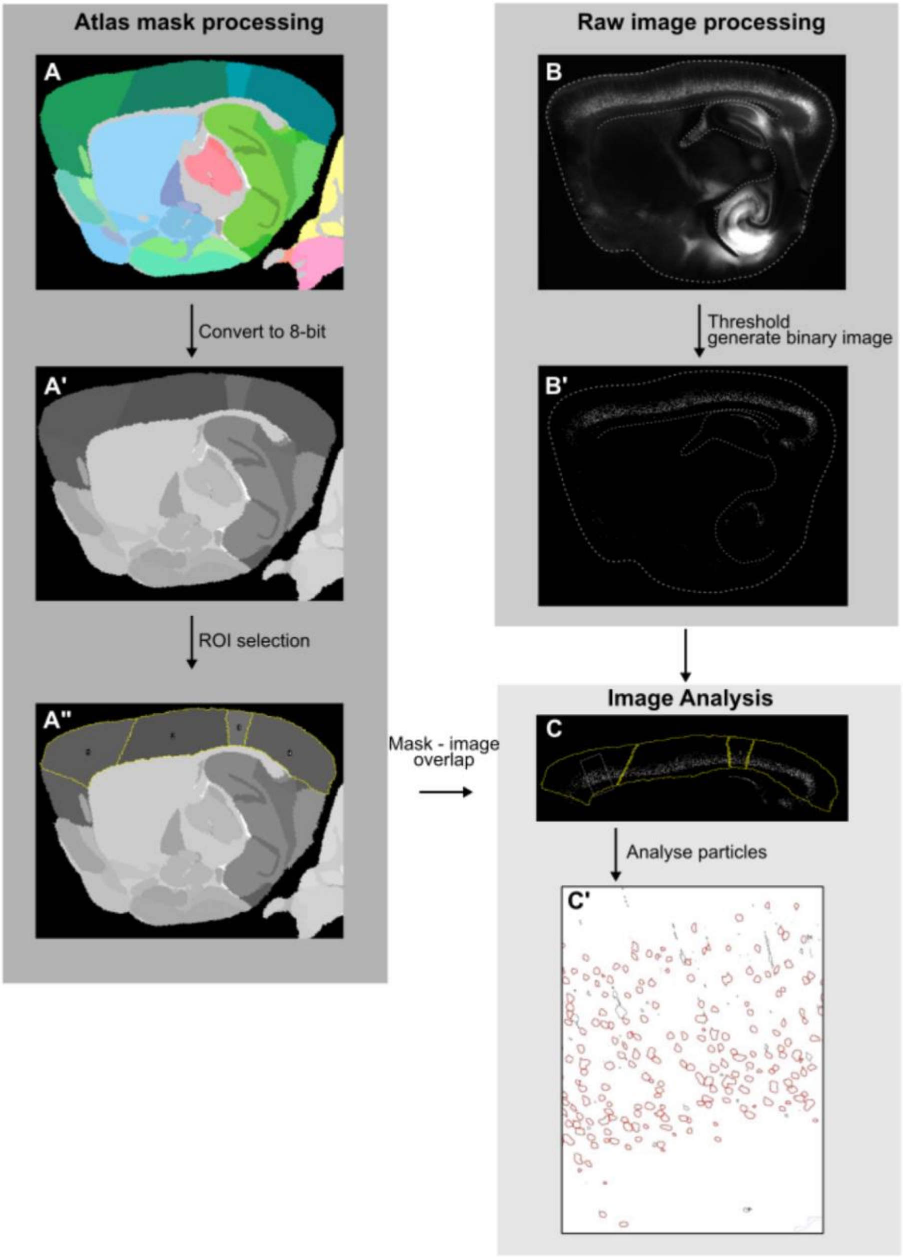

Preliminary steps: create 3 separate folders, one containing the atlas masks (**Panel A**), a second containing the raw TIFF data (**Panel B**) and finally a third folder that will collect all the output images and excel files.

Macro steps: from here onwards, every phase is automatically applied to each pair of segmentation mask/raw file.

1. Resizing of the atlas mask to the same size (dimension) as the raw TIFF image, bilinear interpolation.
2. Superimposing of the resized mask to the raw TIFF image

© 2022. Published by The Company of Biologists under the terms of the Creative Commons Attribution License (https://creativecommons.org/licenses/by/4.0/).

3. Conversion of the resized mask image to 8-bit greyscale (**Panel A'**)
4. Selection of the areas of interest by thresholding (**Panel A''**) (each area of interest is characterized by a specific and unique grey value, i.e.: motor areas, 93; somatosensory areas, 84; auditory areas, 110 and visual areas 94).
5. Background subtraction on the raw TIFF image to eliminate unspecific signal
6. Thresholding to determine the specific signal and binarize the image (Yen thresholding method, value 58) (**Panel B'**)
7. Application of "Watershed" filter to cut out large, sticky spots, avoids clusters/aggregates of spots
8. For each ROI of interest: counting the number of spots via Analyze Particles with a circularity parameter between 0.5 and 1 (to keep only the roundest spots/cells) and a size between 50 and 1500 pixels^2. All elements outside these criteria are rejected. (**Panel C**)
9. Masks corresponding to the selected features of interest are automatically saved in the output folder, under the name "XCountingY", where X is the rank of the analyzed image and Y is the ROI number.
10. A summary Excel file with the 4 ROIs analyzed for each image is automatically saved in the same folder.

References:

Alfano, C., Magrinelli, E., Harb, K., Hevner, R. F. and Studer, M. (2014a). Postmitotic control of sensory area specification during neocortical development. *Nature communications* **5**, 5632.

---- (2014b). Postmitotic control of sensory area specification during neocortical development. *Nature communications* **5**, 5632.

Alfano, C., Magrinelli, E., Harb, K. and Studer, M. (2014c). The nuclear receptors COUP-TF: a long-lasting experience in forebrain assembly. *Cellular and molecular life sciences : CMLS* **71**, 43-62.

Armentano, M., Chou, S. J., Tomassy, G. S., Leingartner, A., O'Leary, D. D. and Studer, M. (2007a). COUP-TFI regulates the balance of cortical patterning between frontal/motor and sensory areas. *Nature neuroscience* **10**, 1277-1286.

---- (2007b). COUP-TFI regulates the balance of cortical patterning between frontal/motor and sensory areas. *Nat Neurosci* **10**, 1277-1286.

Bertacchi, M., Parisot, J. and Studer, M. (2019). The pleiotropic transcriptional regulator COUP-TFI plays multiple roles in neural development and disease. *Brain Res* **1705**, 75- 94.

Bertacchi, M., Romano, A. L., Loubat, A., Tran Mau-Them, F., Willems, M., Faivre, L., Khau van Kien, P., Perrin, L., Devillard, F., Sorlin, A., et al. (2020). NR2F1 regulates regional progenitor dynamics in the mouse neocortex and cortical gyrification in BBSOAS patients. *EMBO J*, e104163.

Gu, Z., Kalambogias, J., Yoshioka, S., Han, W., Li, Z., Kawasawa, Y. I., Pochareddy, S., Li, Z., Liu, F., Xu, X., et al. (2017). Control of species-dependent cortico-motoneuronal connections underlying manual dexterity. *Science* **357**, 400-404.

Harb, K., Magrinelli, E., Nicolas, C. S., Lukianets, N., Frangeul, L., Pietri, M., Sun, T., Sandoz, G., Grammont, F., Jabaudon, D., et al. (2016). Area-specific development of distinct projection neuron subclasses is regulated by postnatal epigenetic modifications. *eLife* **5**.

Leergaard, T. B., Alloway, K. D., Mutic, J. J. and Bjaalie, J. G. (2000a). Three-dimensional topography of corticopontine projections from rat barrel cortex: correlations with corticostriatal organization. *J Neurosci* **20**, 8474-8484.

Leergaard, T. B. and Bjaalie, J. G. (2007). Topography of the complete corticopontine projection: from experiments to principal Maps. *Front Neurosci* **1**, 211-223.

Leergaard, T. B., Lakke, E. A. and Bjaalie, J. G. (1995). Topographical organization in the early postnatal corticopontine projection: a carbocyanine dye and 3-D computer reconstruction study in the rat. *J Comp Neurol* **361**, 77-94.

Leergaard, T. B., Lyngstad, K. A., Thompson, J. H., Taeymans, S., Vos, B. P., De Schutter, E., Bower, J. M. and Bjaalie, J. G. (2000b). Rat somatosensory cerebropontocerebellar pathways: spatial relationships of the somatotopic map of the primary somatosensory cortex are preserved in a three-dimensional clustered pontine map. *J Comp Neurol* **422**,

© 2022. Published by The Company of Biologists under the terms of the Creative Commons Attribution License (https://creativecommons.org/licenses/by/4.0/).

246-266.
Mihailoff, G. A., Adams, C. E. and Woodward, D. J. (1984). An autoradiographic study of the postnatal development of sensorimotor and visual components of the corticopontine system. *J Comp Neurol* **222**, 116-127.
O'Leary, D. D. and Terashima, T. (1988). Cortical axons branch to multiple subcortical targets by interstitial axon budding: implications for target recognition and "waiting periods". *Neuron* **1**, 901-910.
Porrero, C., Rubio-Garrido, P., Avendano, C. and Clasca, F. (2010). Mapping of fluorescent protein-expressing neurons and axon pathways in adult and developing Thy1-eYFP-H transgenic mice. *Brain Res* **1345**, 59-72.
Tomassy, G. S., De Leonibus, E., Jabaudon, D., Lodato, S., Alfano, C., Mele, A., Macklis, J. D. and Studer, M. (2010). Area-specific temporal control of corticospinal motor neuron differentiation by COUP-TFI. *Proc Natl Acad Sci U S A* **107**, 3576-3581.

Original submission

First decision letter

MS ID#: DEVELOP/2021/200026

MS TITLE: Topography of corticopontine projections is controlled by postmitotic expression of the area-mapping gene Nr2f1

AUTHORS: Chiara Tocco, Martin Ovsthus, Jan G Bjaalie, Trygve Brauns Leergaard, and Michele Studer

Thank you for submitting your manuscript via Review Commons to Development. I have read the manuscript along with the reviewers comments and I have consulted with another editor. All manuscript files can be accessed online: please go to BenchPress and click on the 'Manuscripts with Decisions' queue in the Author Area.

We agree that understanding the role of cortical patterning genes in regulating cortical projections is of interest to the field and identifying a direct involvement of Nr2f1 in the topography of corticopontine projections is a novel finding. We also recognise that the study is of a high quality, The three referees of this paper have somewhat divergent opinions. We agree that the revisions you propose should satisfactory address the main criticisms they raise. We would therefore encourage you to revise the manuscriptsalong the lines you have suggested. I will then be happy receive a revised version of the manuscript. Your revised paper will be re-reviewed by one or more of the original referees, and acceptance of your manuscript will depend on your addressing satisfactorily the reviewers' major concerns. Please also note that Development will normally permit only one round of major revision.

We are aware that you may be experiencing disruption to the normal running of your lab that make experimental revisions challenging. If it would be helpful, we encourage you to contact us to discuss your revision in greater detail. Please send us a point-by-point response indicating where you are able to address concerns raised (either experimentally or by changes to the text) and where you will not be able to do so within the normal timeframe of a revision. We will then provide further guidance. Please also note that we are happy to extend revision timeframes as necessary.

Please attend to all of the reviewers' comments and ensure that you clearly highlight all changes made in the revised manuscript. Please avoid using 'Tracked changes' in Word files as these are lost in PDF conversion. I should be grateful if you would also provide a point-by-point response detailing how you have dealt with the points raised by the reviewers in the 'Response to Reviewers' box. If you do not agree with any of their criticisms or suggestions please explain clearly why this is so.

© 2022. Published by The Company of Biologists under the terms of the Creative Commons Attribution License (https://creativecommons.org/licenses/by/4.0/).

First revision

Author response to reviewers' comments

Reviewer #1 (Evidence, reproducibility and clarity (Required)):

Tocco et al examine the changes in the topographic organization of corticopontine projections and infer the ratio of corticopontine versus corticospinal projections from motor and somatosensory cortex after knocking out the expression of Nr2f1 at two developmental timepoints. They conclude that Nr2f1 acts as a molecular determinant of the ratio between corticopontine and corticospinal projections at the progenitor stage and somatosensory corticopontine topographic organization at postmitotic developmental stages. The study is very well written and the Figures are of high quality. The justification for many experimental steps could be clarified, and some are not clear until the discussion. The early Figures, although nice, seems somewhat tangential and equally the authors could instead add an investigation of the Emx1-cKO mice using anterograde tracer injections as in Figure 7 to assess the contribution of S1 directly to the increased corticopontine labelling. The dynamics of the KO model in combination with the utilization of the Thy1-YFP mouse line were not clear to me and could be improved to increase accessibility to a less specialized audience.
→***Authors response*:** *We thank the reviewer for considering our study well-written with high quality figures. As recommended, we have made substantial efforts to clarify the issues raised, and justified why we did not include the tracing part of the Emx1-cKO mouse model. Our point- by-point responses are given below.*

Major comments:

1. Why do the authors investigate only somatosensory and motor regions - especially given the high expression of Nr2fl in the caudolateral cortical regions? For instance, a comparison could have been made to the visual cortex from the start.
→***Authors response*:** *Thank you for the interesting and relevant question. We were primarily interested in the topography of corticopontine projections related to cerebellar functions with tactile representations and the majority of those originate from the somatosensory (S1) cortex. In rats, the somatosensory projections of the cerebro-cerebellar system constitute the largest proportion of the system, with highly organized projections to central parts of the pontine nuclei (Leergaard et al., 2000a; Leergaard et al., 2000b), while projections from the visual cortex are located in the lateral and rostral periphery of the pontine nuclei, and therefore not very amenable for distinguishing changes in topography. In addition, our previous work on Nr2f1 showed dramatic changes in the S1 area which takes a motor-like identity upon loss of Nr2f1 (Alfano et al., 2014b; Armentano et al., 2007b). So, while it would be interesting to assess impact of Nr2f1 on topographical organization of several subcortical projection systems, we deemed more logical to first investigate impact on the topographic organization in somatosensory projections in comparison with projections from the motor cortex. This is emphasized in our revised text on page 6 (lines 151-156).*

2. My instinct is that Figure 1 could be supplementary (if needed at all). It may be nice to have in case someone wants to consult the specific methodology after reading the main results but is not extensively referred to in the text and is overly complicated for a quick understanding of the experimental design. The flow and justification should be made clearer in the texts as one goes. Also, it is indicated in the flow chart that the Allen brain atlas data is only used for Figure 2 - however, Figure 3 figure legend also claims to be data from Allen. I assume this Figure labels in this schematic are wrong.
→***Authors response*:** *We thank the reviewer for pointing out the mistake in Figure 1. We have now simplified the flowchart of the different processing and analytic steps and adapted it to some of the figures in relation to the type of experimental approach. We consider that it is important to keep this corrected workflow as Figure 1, so that the reader can easily go back to it. In this way single procedures can be referred in the text, and the workflows should be easier to follow when reading the main text and figures.*

3. Similarly, while figure 2 is quite a nice figure, the significance of this comparison is more of a

© 2022. Published by The Company of Biologists under the terms of the Creative Commons Attribution License (https://creativecommons.org/licenses/by/4.0/).

methodological control rather than a result.
→***Authors response*:** *The purpose of this figure was twofold. On one side, we wished to present the published data from the Allen Mouse Brain Connectivity Atlas used as supplementary controls for one of our tract tracing paradigms; on the other side, we aimed to demonstrate that the 3D point data derived from tract tracing experiments in wild-type mice from the Allen institute, and our control animals yielded comparable results. We have therefore followed the reviewer's advice and combined Figures 2 and 3 into a new Figure 2, which exclusively presents the 3D visualization of topographical organization based on tract tracing data from the Allen Mouse Brain Connectivity Atlas (see also below).*

Figure 3 is also a nice confirmation of corticopontine projections between mice and rats; although this is slightly more comprehensive and direct than what has been previously shown for S1 and M1/M2 (in studies cited by the authors), the amount of text and Figure space dedicated to this point seems excessive as it is not the main point or novel finding of the study. Perhaps Figure 2 and 3 could be combined and the point succinctly made with respect to the Allan brain atlas data with a confirmation from the lab's injections sites as a supplement. It seems to me that the entire point can be well made with the text from lines 184-189 alone.
→***Authors response*:** *We agree that the demonstration of topographical organization in the mouse pontine nuclei is a secondary point of the study, but we consider it very important to include, since our demonstration that the topographical distribution patterns in mice resembles those demonstrated earlier in rats is a novel finding, and essential to the interpretation of our tract tracing results in Nex-cKO mice. We have therefore followed the Reviewer's advice and combined Figures 2 and 3 (now Figure 2), with downscaled panels and reduced the descriptions in the main text substantially.*

4. Lines 221-230: The justification for examining both lines (and embryonic ages) is not fully clear. The intro (lines 94-102) also does not indicate why these two lines (representing developmental stages E9.5 and E11.5) are hypothesized to be interesting to compare.
→***Authors response*:** *We apologize for this confusion and have added a few statements at pages 5 in the Introduction, 6 in the Result section and, 15 in the Discussion part for clarifying the reason of using two independent conditional lines. In brief, the Emx1-cKO line challenges the role of Nr2f1 in cortical progenitors and postmitotic progeny (thus neurons) by acting early during development (at E9.5), while the Nex-cKO line challenges Nr2f1 function exclusively in postmitotic neurons (at E11.5), since normal expression is maintained in progenitors. This specific difference helps us distinguishing whether Nr2f1 acts at the level of progenitor cells or differentiating neurons during establishment of corticopontine topography.*

5. The strategy of using the Thy1 reporter mice means that you are only seeing effects of the KO in a sub-population of selected neurons. Clearly, this was desired to focus on the corticospinal neurons, but this is not adequately explained. This also relies on the specification of this population for this purpose. How much heterogeneity is in the expression of the line. Further, this distribution (Figure 4B,B') is counter to the expression levels of Nr2f1 (Figure 7a). Is this of significance and why did the authors choose this approach? Line 258: 'as assessed by the YFP signal expression': but what is the logic for Thy1-driven YFP to be altered by Nr2f1? Although this is mentioned briefly in lines 226-229, this could be made more direct.
→***Authors response*:** *It is correct that our aim was to use a reporter line expressed only in subcerebral projection neurons of layer V. The Thy1-eYFP line is perfectly suitable for this approach and is reported as a reliable and reproducible layer V reporter line in several studies. There is no particular heterogeneity reported in this line, although expression levels can slightly change between animals. Expression differences may also be ascribed to minor variations in the experimental fixation procedures. The YFP expression follows the physiological distribution of subcortical layer V projections, with a high density in motor cortex and a gradually decreased distribution in S1 and more caudal areas (Porrero et al., 2010). Since the distribution of layer V neurons changes in the absence of Nr2f1, as previously reported in our studies (Alfano et al., 2014c; Armentano et al., 2007a; Tomassy et al., 2010), the distribution of Thy1-YFP signal will consequently be altered as a read-out of layer V changes. We thus asked whether these alterations in layer V distribution observed in the two mutant lines would be also reflected in layer V axons projecting towards the pons. It turned out that this is the case. For example, high density of layer V neurons in S1 (indicated as a higher number of YFP+ neurons) is translated in*

© 2022. Published by The Company of Biologists under the terms of the Creative Commons Attribution License (https://creativecommons.org/licenses/by/4.0/).

an abnormally higher corticopontine innervation. We realize that our paradigm was insufficiently explained, and to clarify this we have revised the Result section (pages 7,8).

6. It is also unclear why the expression of YFP shifts to be more highly expressed in caudal regions in the KO lines. The pattern of results if just M1/M2 and S1 are taken into account makes sense (e.g. as summarized in Figure 8), but what is the implication for increased YFP expression in caudal regions? Is the increase in pontine labelling from just increased YFP signal in caudal regions or a true trade-off between CST and CP pathways? I am missing a clear explanation of the role of Nr2f1 in the designation of corticopontine fibers. If low gradient of Nr2f1 results in more corticospinal projections (e.g. in motor cortex) in controls, then knocking out Nr2f1 in progenitor cells results in more corticopontine fibers? But realistically what is being measured is more Thy1-driven YFP expression caudally, which may just highlight cortical regions that already have more corticopontine projections (in relation to corticospinal). For instance, if one adds the Nr2f1 gradient information to Figure 8, It is not clear to me what the direct relationship is here.
→***<u>Authors response</u>:*** *We fully appreciate the reviewers question and apologize for insufficient explanation of our experimental paradigm. We used the Thy1-H-eYFP line as means to label exclusively layer V projection neurons (corticospinal and corticopontine) and investigate the corticopontine axonal projection topography. We expected that an abnormal cortical distribution of YFP-expressing layer V neurons would be translated into altered corticospinal projections and/or corticopontine topographic mapping. This is the reason why we first assessed the spatial organization of YFP-expressing layer V cortical distribution in mutant adults compared to control animals. Since the YFP reporter line labels layers V projecting neurons across the entire cerebral cortex, high caudal expression of YFP (particularly in the visual cortex) implies a higher density of layer V neurons in this area (mainly corticotectal projection neurons). We have mentioned this phenomenon in our recent paper (Bertacchi et al., 2020), showing that in the absence of Nr2f1, progenitors in the caudal cortex tend to proliferate more and to produce a higher number of deep layer neurons. Since the present study focuses on motor and somatosensory cortices for the reasons mentioned above, we have not followed the trajectory of this excess of layer V projections neurons in the visual areas. This might be very interesting with respect to retino-geniculate topography, which is not the topic of this study. Finally, the gradient of Nr2f1 plays distinct roles along the cortex, as described in our recent review (Bertacchi et al., 2019). This implies that in its absence, S1 identity (where Nr2f1 is expressed) is not properly specified and motor identity (where Nr2f1 is not expressed) takes over since not repressed by Nr2f1 in S1. In addition, overexpression of Nr2f1 in motor cortex leads to a change in sensory identity, as shown by markers and thalamocortical projections (Alfano et al., 2014a). In brief, Nr2f1 is necessary and sufficient to impart sensory identity in the developing neocortex. To better explain our paradigm, we have revised the Introduction (pages 4, 5), where we also point to our earlier findings.*

7. Related to above, why was Figure 7 only done in Nex-cKO mice? It would be informative to see the direct effects of the area specific projections in the Emx1-cKO in this regard.
→***<u>Authors response</u>:*** *This is a legitimate question, and we apologize for not having better clarified the choice of the Nex-cKO model. The main reasons are now explained at the beginning of the paragraph dealing with the viral injection experiments. Since we found unexpected aberrant and homogenous corticopontine projections in the Emx1-cKO line, we rationalized that tracing the cortical origin of all the projections would not give us a clear answer on topographic shifts, by making the study of topographic mapping very difficult to interpret. We thus decided to focus on the Nex-cKO mice, which topographical shifts was already better defined with the help of the reporter Thy1-YFP, and therefore more suitable for experimental area-specific tract tracing studies.*

8. In addition, some examples of actual injections sites (even in a supplementary Figure) would be helpful here since the mice were injected at P5, how does this affect the injection site volume/spread etc compared to the Allan brain data.
→***<u>Authors response</u>:*** *We fully agree with the reviewer that transparency and full documentation tracer injection sites are important. To improve the documentation of our experimental material, we have added microscopic images documenting representative injection sites and pontine labelling to new Figures 6 and 7 (previously Figure 7) and added 3 <u>new supplementary figures (Suppl. Figures 3 to 5)</u> showing the centres of all injection sites, together with representative*

© 2022. Published by The Company of Biologists under the terms of the Creative Commons Attribution License (https://creativecommons.org/licenses/by/4.0/).

documentation of the pontine labelling. At P5, the motor and somatosensory cortical areas are well defined and amenable to inject at the expected locations. In addition, we will also share the complete collection of microscopic images via the EBRAINS infrastructure (http://ebrains.eu).

9. The semi-quantitative assessment in Figure 4F is confusing. Why not put regions of interest around the various areas and get a quantitative measure of layer 5 intensity? Or ideally, use image analysis tools for cell detection. The images seem like they would be suitable for this.
Especially if the authors wish to do statistics based on this data, a less subjective and more quantitative measure is certainly required and not difficult to do. Additionally, the current scale is described as going from 0-4, but the mean is sometimes >4. So, did the scale actually go to 5? I appreciate the amount of work that went into this especially with regard to Supplementary Figure 4, but the subjective nature of the method does not seem necessary.
→***Authors response*:** *We thank the Reviewer for pointing out the inconsistency of the numbers in Figure 4F. This is the result of how the analysis was conducted. Previously, every image was aligned with the corresponding Allen brain atlas plate, for area segmentation, and then an intensity score was attributed for each area. We understand now that the intrinsic subjectivity of our previous, more qualitative, analysis could have led to imprecise quantification, and we have now utilized a new automatic Image J macro, which has allowed us to count spots by area of interest in the brain. The ImageJ macro used the find maxima tool to automatically count YFP+ nuclei by area of interest in the brain. The spots have been considered as specific staining using a threshold based on intensity and shape of the elements. This has allowed to estimate the number of YFP-expressing neurons in seven cortical areas (prefrontal, motor, somatosensory, auditory, visual and retrosplenial cortices) defined by delineations derived from spatially registered overlay images from the Allen Mouse Brain Atlas. Our new analysis has resulted in a more reliable analysis of the distribution of YFP positive cells among the different functional areas (new Figure 3). We have also added a detailed pipeline with some examples as an ANNEX at the end of this document (pages 16,17).*

10. Line 288: calling the results an 'impaired cortical distribution of YFP expressing layer V neurons' seems to link to function. Perhaps 'disordered' is a better word here.
→***Authors response*:** *Fully agreed. We have changed it as suggested in our revised manuscript.*

11. Lines 308-312: in Figure 5G and H, the variance across animals is very different and while both show the same trends, the significance changes because of the increased error in G. What is the source of this difference in variance in the measures of rostral and caudal area? This makes the result here difficult to interpret.
→***Authors response*:** *We agree that this is the case, and that the reasons might come from the more severe phenotype of the Emx1-cKO tract that renders the quantification more difficult to assess. However, despite the variance observed, we see a clear trend, and we have now acknowledged this observation in the text at page 9. However, we do not believe that it impacts our overall conclusion.*

12. What is the implication of the increased fasciculation in Figure 5? Lines 322-324: again, functionally speaking, 'controls' seems like a strong word. Can the authors clarify the causal relationship to the fasciculation?
→***Authors response*:** *We agree with the reviewer that we have no direct proof of a causal relationship of Nr2f1 to axonal fasciculation. We have therefore change the sentence to: "…these data show that loss of Nr2f1 expression (both in cortical progenitor cells and postmitotic cells) affects the diameter, shape and degree of fasciculation of the CST originating from layer V neurons. "(page 10).*

13. Much of the useful justification that would make the results easier to follow is in the discussion. I would suggest trying to (as concisely as possible since the results are already quite long) move/summarize some of this justification in key transition periods for the results.
E.g. info from section line 530, also 552.
→***Authors response*:** *We agree that our Results section could benefit from some additional information and have therefore added explanatory text in the Result section, as suggested by the Reviewer's suggestion.*

© 2022. Published by The Company of Biologists under the terms of the Creative Commons Attribution License (https://creativecommons.org/licenses/by/4.0/).

Minor Comments:

- Figure 3. Please outline V1 if you want to indicate it in the figure - since all other areas are outlined, this suggests the entire caudal cortex is V1.
→***Authors response*:** *We thank the Reviewer for pointing this out and have removed annotations for areas not relevant for the present investigations.*

- Figure 3. Please indicate in B/I caudal-rostral, medial-lateral, dorsal-ventral. This will help with easy identification in C-G/J-N.
→***Authors response*:** *This is now Figure 2, to which we have added a new top row which includes 3D renderings of the pontine nuclei, in view from sagittal and ventral, that aid understanding the angle of view of the dot maps shown below. We have added explanation to the Figure legend, and removed the diagonal cross indicating dorsal and ventral relative to the long axis of the brain stem at level of the pons (see also the Reviewers comment to Figure 7, below).*

- Lines 217-221: this is quite a long, complex sentence. Consider rewording.
→***Authors response*:** *We agree and have rephrased using 2 separate sentences.*

- Line 221: might be useful to briefly specify here what control animals are - littermate controls from methods?
→***Authors response*:** *We have now specified that controls are indeed littermate controls.*

- Line 413-414: different font.
→***Authors response*:** *This has been corrected*

- Figure 7: in C, is the left schematic really dorsal (to the top)? According to the guide on the right schematic, would this not be more like rostral? In reality it is more like a R-D angle, but these labels make the 3D views even more difficult to visualize. Perhaps an accompanying schema, perhaps a condensed version of that in Figure 2, would serve useful in the other figures as well.
→***Authors response*:** *The oblique orientation of the brain stem at level of the pons can indeed be confusing. We have therefore edited the orientation annotations and added small images of the Allen Mouse Brain Atlas to make the orientation clearer.*

- What is meant in lines 750-752 that the Franklin and Paxinos atlas was used when sections were matched with the Allen brain atlas? Why was this necessary?
→***Authors response*:** *This is an error related to a previous preliminary analysis, which became obsolete after all images were spatially registered to the Allen mouse brain atlas. The sentence has been deleted.*

- Line 755: what is the P8 in reference to? I only found reference to P21 and adult. Was this for the in situ? This is later specified as P7 (line 768), but P5 in Figure 7 legend (line 468). Please clarify ages.
→***Authors response*:** *We apologize for the confusion and have now corrected the ages.*

- Sometimes they are referred to as Emx-cKO instead of Emx1-cKO.
- Line 721: missing closing bracket.
→***Authors response*:** *We have now uniformed Emx-cKO to Emx1-cKO.*

Reviewer #1 (Significance (Required)):

The study offers a significant advance in our knowledge of how a specific molecular determinant contributes to the specification and organization of corticofugal projections. The authors have also made much of the data available in supplementary Figures and have presented the findings comprehensively. The study represents one of only a handful of such investigations of corticopontine projections in mice, compares in detail to the topography already described in detail in rats, and will be a welcome resource for those studying corticopontine and corticospinal projections in mice. Although the study is very targeted in scope in this regard, the combination of molecular patterning across development and adult anatomical organization means the study will

© 2022. Published by The Company of Biologists under the terms of the Creative Commons Attribution License (https://creativecommons.org/licenses/by/4.0/).

appeal to a wider audience. My expertise is in neuroanatomical and functional aspects of corticofugal projections, not in the molecular patterning across development. In this regard, I have noted a number of areas in which it was unclear to me either the justification for the experimental design or the direct implication of the results. I hope these comments will allow the authors to broaden their target audience even further.
→***Authors response*:** *We thank the reviewer for appreciating the novelty of this study and the importance and advance in the field of topographic control of corticofugal projections.*

Referee Cross-commenting
I also agree with the comments from the other reviewers and echo the major concerns of reviewer 2 in this regard. I will also say that my assessment of 1-3 months for revisions was an optimistic underestimate. I agree that up to 6 months would be more appropriate as the other reviewers suggested.

Reviewer #2 (Evidence, reproducibility and clarity (Required)):

This manuscript from Chiara Tocco, Martin Ovsthus, and colleagues explores the consequences of inactivating the gene Nr2f1/COUP-TFI in cortical progenitors or post-mitotic neurons on the topography of the cortico-pontine projection. The group has extensive knowledge of Nr2f1 and its effects on cortical mapping and laminar development and the consequences of its inactivation on the development of sensory-motor systems. Based on their previous work, the rationale for looking into the role of Nr2f1 on the development of cortico-pontine axons is strong and should yield important information on underexplored aspects of its biology. For this, the authors use 3 complementary approaches: analysis of publicly available tracing data from the Allen Brain Institute, histological analysis of labeled neurons in the Thy1.1-YFP H line crossed with Emx1 and Nex cre lines, and viral tracing in pups in Nex cre lines. Based on these experiments and with the help of alignment and standardization tools for 2d histology, the authors conclude that the post-mitotic absence of Nr2f1 affects the topography of cortico-pontine projections. The two most convincing results presented here are 1) The concentric organization of cortico-pontine projections from the Allen Institute dataset analysis 2) The presence of fasciculation defects following the Emx1 and Nex-cre deletions of Nr2f1.

Otherwise, the methodology used throughout the rest of the manuscript and the presented results do not strongly support the conclusions of this work, as shown in Figure 8.
→***Authors response*:** *We remain confident that our findings of altered organization of corticopontine projections in Emx1-cKO and Nex-cKO brains are robust, but understand that the Reviewer may found discrepancies between our data and summary Figure 8. We have therefore substantially revised the summary panels and placed them in the relevant main figures, the new Figures 5 and 7. A new model has been now added in Figure 8. This summarized our findings with the ones in the literature. We have now clarified the issues pointed out by the reviewer, and adapted the text accordingly, and similarly to Reviewer 1, we have justified why we did not include the tracing part of the Emx1-cKO mouse model. Our point-by-point responses are given below.*

Major points:
1. An important issue is the use of the Thy1.1 H-line reporter to label layer V neurons in the cortex instead of relying more strongly on viral tracings done with stereotaxic injections. The changes in GFP expression patterns both in the cortex or in the Pons in this line crossed with the mutant lines are difficult to interpret because: - The expression of YFP in the cortex is clearly modified in the mutant. Still, it is unclear whether this is due to the absence of layer V neurons, their respecification to other layer types, or merely to differential regulation of the transgene expression in the mutant lines. To answer these questions, the authors could have combined labeling of the YFP with laminar cortical markers and estimate the signal at the single-cell level on sections, to estimate the molecular identity of Thy1.1+ neurons in the mutants. Without precise information on the molecular nature of the labeled Thy1.1YFP neurons in the mutant cortex, it is impossible to interpret potential changes in the distribution of YFP+ fibers in the pons.
→***Authors response*:** *Our major aim was to use a reporter line expressed only in layer V neurons and follow the subcortical projection pattern along the whole cortex before focusing on the somatosensory and motor cortices, known to massively contribute to the pontine nuclei*

© 2022. Published by The Company of Biologists under the terms of the Creative Commons Attribution License (https://creativecommons.org/licenses/by/4.0/).

innervation. We consider that the Thy1-H line is perfectly suitable for this approach and is reported as a reliable and reproducible layer V reporter line in several studies. There is no particular heterogeneity reported in the Thy1-H line, although expression levels can slightly change between animals. Expression differences may also be ascribed to minor variations in the experimental fixation procedures.

It is well reported that the YFP expression follows the physiological distribution of subcortical layer V projections, with a high density in motor cortex and a gradually decreased distribution in S1 and more caudal areas (Porrero et al., 2010). Since the distribution of layer V neurons changes in the absence of Nr2f1, as previously reported in our studies (Alfano et al., 2014c; Armentano et al., 2007a; Tomassy et al., 2010), the distribution of the Thy1-YFP signal will consequently be altered as a read-out of layer V changes. We thus asked whether these alterations in layer V distribution observed in the two mutant lines would be also reflected in the organization of their projections toward the pontine nuclei. It turned out that this is the case. For example, high density of layer V neurons in S1 (indicated as a higher number of YFP+ neurons) is translated into an abnormally higher and confused corticopontine innervation.

We acknowledge that combined labelling of the YFP with laminar cortical markers, would give us more information on the molecular identity of Thy1-YFP+ neurons in the mutants. But this is not really the focus of this study, which instead was in part answered in our previous reports (Alfano et al., 2014b; Harb et al., 2016; Tomassy et al., 2010). Here, we are mainly interested in understanding whether proper cortical area mapping is involved in corticopontine topography since very little is known on the role of molecular determinants controlling corticopontine connectivity. Therefore, we consider that using a mouse model in which cortical area mapping is perturbed very early during development (without affecting subcerebral structures and including the pontine nuclei) is perfectly suitable for validating our initial hypothesis of a spatio-temporal establishment of area-specific targeting of corticopontine neurons.

2. The density of the labeling in the Pons is extremely high, and the phenotypes showed in figure 6 are not clear. It is appreciated that the authors included a complete survey of the cases in the supplementary information, but this highlights the strong variability in the precise signal distribution across cases and the phenotypes are not convincing. An unbiased quantification with statistics of the signal distribution could have helped. Because fibers from the whole cortex are labeled, this experiment doesn't allow to conclude on the effect of the mutation on the pontine projections, apart from the fact that they are disorganized in both mutants (Fig. 5).
→***Authors response*****:** *The density of fluorescence expression in the pontine nuclei shown in Figure 6 (now Figure 5) and extensively documented in Supplementary Figs. 1 and 2, is variable across cases in terms of signal intensity, but not in terms of innervation patterns. Our microscopic images and semiquantitative analysis therefore focussed on the spatial distribution of signal expression, rather than on quantification of the signals. But, the collections of spatially corresponding images shown in Supp. Figure 2 clearly show reduced signal expression in central parts of the pontine nuclei in all Nex-cKO cases, compared to the controls. We maintain that these findings give strong indications, which are further supported by the subsequent tract tracing data.*

3. Likewise, it is not easy to interpret differences in the size of the labelled CST tract, as it could be due to either guidance defects upstream, loss of layer V neurons, loss of YFP expression in the mutants, or all of the above, so the experiment shown in Fig 5 is potentially interesting, but not informative on its own and should be combined with a complete survey of the tract in the rest of the brain, and complemented with tracing methods.
→***Authors response*****:** *We appreciate the reviewer's comment, however Figure 5 (now Fig.4) shows that the diameter of the fascicle at the level of the cerebral peduncle is not changed among groups (also quantified in panel G), which excludes any important loss of neurons or YFP expression in the mutant groups. What this figure shows is a different contribution of axons towards either the pontine nuclei (increased in the Emx1-cKO group but maintained in the Nex-cKO one) and a fasciculation defect past the pontine nuclei in both groups. A complete survey of the tract is a very interesting suggestion but could not be part of this study, since out of focus.*

4. maybe a typographic error, but mouse number 18166 appears twice as both a mutant and control in the supplementary information.

© 2022. Published by The Company of Biologists under the terms of the Creative Commons Attribution License (https://creativecommons.org/licenses/by/4.0/).

***Authors response*:** *We acknowledge the mistake in the supplementary figure 1 and confirm that it was indeed a typographic error. As reported in supplementary table 2, animal #18166 is a control case, whereas the correct number of the third Emx1-cKO animal is in fact the #18046. The numbers have been corrected in the new version of the figure.*

There are also issues with the **viral tracing experiments**:

4. The drawn maps for the injection assume a wild-type map, which is not the case in the mutants for area sizes. Therefore, the injections in the mutant brains may not be in the actual region shown. Also, the map doesn't include multi-sensory integrative regions, which have an extremely different connectivity pattern than sensory-motor regions. As a result, the sizes of V1, S1, and A1 are too large, as these integrative regions occupy territories in between, and some injection points may fall in one of these regions.
→***Authors response*:** *The serial histological sections were carefully registered to the 3D reference atlas using non-linear adjustments, based on multiple anatomical landmarks. The injection site locations were mapped onto the reference atlas using 3D spatial coordinates (and not area identity) to allow comparisons of spatial the locations of injection site centres. The cortical areal identities are certainly changed in the mutants, this is key to the experimental paradigm of our study. The spatial accuracy of our methodology is validated in Figure 2 (and Supp. Figs. 3-5), which shows similar spatial distributions of data points representing corticopontine labelling obtained by independent tract-tracing studies in wild- type (Allen institute data) and our control experiments. Interestingly, our tracer injections in control experiments yield similar results as those in wild-types, which also strongly resemble our earlier findings reported in rats (Leergaard and Bjaalie, 2007). The area designation of the atlas is only used to indicate assumed motor or somatosensory area identity, as only the spatial coordinates were used to identify spatially corresponding experiments for our comparisons. The annotation labels for A1 and V1 have been removed.*

5. The size of the injection spot shown may be misleading: considering the considerable amount of virus injected in the first post-natal week (500nL to 1µL at 10^12!), it is likely that the labeled neurons are located in a much larger area than shown on the figure. The authors should provide the actual histological data in the injection sites to support the figure and the precision of the injection point. Moreover, raw images of the pontine projections would be welcome on top of the segmented images shown.
→***Authors response*:** *We fully agree that full documentation of tracer injection sites is essential. All suitable microscopic image data will be publicly shared but following the Reviewer's advice we have now also added microscopic images of representative injection sites and pontine labelling to the new Figures 6 and 7 (previously Fig. 7) and Supple. Figs. 3 to 5. These images demonstrate that the tracer injections are spatially delimited with a relatively small extent.*

6. It is difficult to parse the mutant phenotype from the results of the viral tracings: control injections in the somatosensory map show widely different projection patterns: panels G, H, and I have close injection sites in controls, but very different projection patterns. Could this be due to registration errors? Also, figure 3 suggests that the mediolateral location of the neuron is an important factor in determining pontine topography. Maybe a color map as in Figure 3H-N would be more informative to compare mutants and controls. However, in the current state, the variability of injection results in controls and mutants alike would prevent the reader from drawing strong conclusions on the phenotype.
→***Authors response*:** *The tract tracing experiments illustrated in previous Figure 7 (panels F- I, but not panel J) very clearly show that tracer injections in somatosensory areas in Nex-cKO brains give rise to substantially different projection patterns compared to spatially corresponding injections in control brains. These differences in spatial distribution are very distinct, in particular for tracer locations in medial parts of S1. Given our newly achieved understanding of corticopontine topography in the mouse brain (cf. our analysis of Allen Mouse Brain Connectivity data) and our findings that tracer injections in control animals yield the same labelling patterns as public data for wild-types, the differences observed in the mutants cannot be ascribed to registration errors. We realize that Figure 7 was very information rich and have therefore split it in two new figures (now Fig. 6 and 7), where Figure 6 demonstrates how tracer injections in motor cortex of mutants give similar results as injections in controls/wild-types, while Figure 7 shows how projections from somatosensory regions in mutants are very different from controls.*

© 2022. Published by The Company of Biologists under the terms of the Creative Commons Attribution License (https://creativecommons.org/licenses/by/4.0/).

7. Finally, as the claim is that Emx1 cre based deletion of Nr2f1 has a different phenotype, injections in this background would be mandatory to draw this conclusion.
→***Authors response*:** *As mentioned above for Reviewer 1, we decided not to do the tracing tract experiments in the Emx1-cKO line, because our Thy1-YFP analyses (now Fig. 5) showed an unexpected aberrant and homogenous corticopontine projections in these mutants, differently from the Nex-cKO line in which the shifts were more clear-cut. We think that tracing the cortical origin of all the projections in these mutants will fail to give us a clear answer on topographic shifts by making the study of topographic mapping very difficult to interpret. We thus decided to focus on the Nex-cKO mice, which topographical shifts was already better defined, and therefore more suitable for experimental area-specific tract tracing studies. It is however implicit that topographical shifts are embedded in the phenotype of the Emx1-cKO brains, but because of the abnormal increase of corticopontine innervation in theses mutants, this shift becomes very difficult to discern.*

8. The results presented in Figures 3 and 7 don't strongly support the summary of figure 8. Figure 3 shows that the mediolateral location of the neuron is the main factor that drives the topography of the Pons, as sensory and motor projections (likely originating from a similar lateral position) overlap in the caudal pons, a fact not represented in the summary on Figure 8. Moreover, since viral injections have not been done in the Emx1 mutant, the authors cannot conclude on the organization of these projections based solely on the results of figure 6, which are not precise enough to assess a normal topography.
→***Authors response*:** *We agree that our summary diagram in Figure 8 was not reflecting the results observed in Emx1-cKO mice and have therefore made separate diagrams summarizing findings achieved with our different paradigms (Fig. 4 and 5, respectively). The revised diagrams now better capture the main findings observed in our material. We decided instead to add a final summary figure (Fig. 8) that also includes data from the literature and illustrates that both, the cortex and pontine nuclei, are involved in corticopontine topography mapping.*

9. To obtain a better description of the phenotype of both mutants, the authors should have relied on viral mapping done in adult animals, where it is possible to carry precise stereotaxic injections of smaller volumes (10-50nL) of virus in the cortex. It would have been possible also to use dual-injections strategies to label cortico-pontine projecting neurons specifically. Unfortunately, the methodology presented here only gives partial and hard to interpret data.
→***Authors response*:** *Topographically organized corticopontine projections are established early in rodents (Leergaard et al., 1995; Mihailoff et al., 1984; O'Leary and Terashima, 1988), and injections at post-natal stages are normally accepted (e.g., Gu et al., 2017). Juvenile mice at P21 will thus display the same topographical projections as in adults, if anything slightly more widespread than in adult animals. Finding altered projection topography in young animals only strengthens our conclusions. As commented above, our tracer injection sites are very delimited and relatively small, as shown in new Fig. 6 and 7, and in our new Supplementary Figures 3 to 5, showing overview of all injection sites. We believe that we have now provided enough documentation showing that our tract tracing results in control animals give similar results as shown in the public data collection of the Allen Institute, and that the topography of corticopontine projections are altered in mutant animals receiving tracer injections in somatosensory areas, but not in animals receiving tracer injections in motor areas.*

Minor points:

- Figure 3: the medio, lateral and anterior axis should be shown in panels B-G and I-N
→***Authors response*:** *We have rearranged the Figure (now Fig. 2) and added annotations indicating orientation for all panels.*

- Figure 4: fully quantitative count of the neurons should be done, instead of the qualitative (not "semi-quantitative"!) scale used here.
→***Authors response*:** *We have completely revised our quantification approach for assessing the density of YFP expressing neurons in the cerebral cortex, as illustrated in the new figure (Fig. 3). Detailed description has been added on pages 20-21 and in the figure legend.*

© 2022. Published by The Company of Biologists under the terms of the Creative Commons Attribution License (https://creativecommons.org/licenses/by/4.0/).

- Figure 5: can you indicate the standard deviation in the curves E, F?
→**_Authors response_**: *We understand the concern of the reviewer, but we think that the addition of standard deviation curves to panels E and F might affect the overall readability of the graphs. However, the reader can consult the complete summary of statistical analysis performed in this work, reported in supplementary table 4.*

- Methods: why is affine transformation done and not deforming transformation? This may lead to a more accurate alignment.
→**_Authors response_**: *The spatial registration of images was indeed optimized using a secondary non-linear registration step with the tool VisuAlign, as mentioned several times in the Methods text. However, in the main description of the spatial alignment procedure in the Methods section, this description was missing. We have now added the missing text (lines 678- 681).*

Reviewer #2 (Significance (Required)):

This study carries the work done by the group on the importance of post-mitotic expression of areal-specification genes. While a lot has been done on the role of COUP-TFI on cortical development, it is important to assess the consequences mapping errors can have on the topography of projections and how genes controlling the nature of cortical areas can also act directly on axon guidance. While this has been done in other contexts, the focus on the post-mitotic role of these genes here is noteworthy and interesting.
→**_Authors response_**: *We thank the reviewer for appreciating the importance of post-mitotic expression of areal-specification genes in topographic mapping during cortical development.*

Referee Cross-commenting

I agree with the comments from other reviewers, some of which confirm also my major concerns that 1) More precise analysis with viral tracings are warranted and 2) The use of the Thy1.1-YFP reporter in a mutant context makes it difficult to interpret the results.

Reviewer #3 (Evidence, reproducibility and clarity (Required)):

In this manuscript, Tocco et al investigate a possible role of Nr2f1 expressed in cerebral cortical neurons in regulating the topography of corticopontine projections. The authors employ two different conditional deletions of Nr2f1 to delete the gene in precursors or postmitotic neurons. By crossing these animals with a Thy-YFPO reporter line they can quantify the number of corticospinal L5 neurons and describe the topography of their projections in the pontine nucleus. This is a beautiful approach to investigate if intrinsic information in cortical progenitors and postmitotic neurons determines subcortical connectivity.
→**_Authors response_**: *We thank the reviewer for considering our study a beautiful approach to understand whether and how intrinsic cortical information influences subcortical connectivity.*

The first set of experiments show that Nrf21 determines area-specific distributions and total numbers of L5 corticospinal neurons. In general, the expression of YFP show decreases in rostral areas and increases in caudal regions. When Nr2f1 is deleted in postmitotic neurons the net result seems an overall reduction of the total number of YFP expressing neurons. Deletions in precursors cells change the distribution, and not so much the numbers of YFP neurons in the cortical areas. The authors conclude that Nrf2 plays different roles in the differentiation of L5 precursors and postmitotic neurons. Quantifications of these experiments are adequate and conclusions are correct although not surprising taking into account previous work from the group.
In the second part of the study, the authors analyze the topography of corticospinal and corticopontine projections in mutants in which Nrf21 is genetically deleted to investigate if the different cortical distribution and presumably a modified identity, correlate with distinct mapping. They perform viral injections and beautiful image reconstructions. This is an elegant approach. The main obstacle here is that the descriptions of the distribution of projections is qualitative and not quantitative. The number of injections is also restricted to two or three injections per area. With

© 2022. Published by The Company of Biologists under the terms of the Creative Commons Attribution License (https://creativecommons.org/licenses/by/4.0/).

those numbers and without quantifications the study does not allow to describe the variability of the experimental method and more importantly, the intrinsic variability of projections within each of the cortical areas tested. This is an important obstacle for any strong conclusions. In WT animals, for example, the results suggest rather noisy maps.
→***Authors response*:** *We thank the reviewer for evaluating our image reconstruction work as an elegant approach. Below, we have responded to the reviewer's concern regarding quantifications.*

Major concerns:

1. The lack of quantifications and the limited number of injections make the study more descriptive than conclusive.
→***Authors response*:***The reviewer correctly observes that there are variations in the amount and spatial distributions across tract tracing experiments. This well-known variation is closely related to the location and extent of the tracer injection sites. The exact zone of tracer uptake varies with type of tracer and application method and is very difficult to assess quantitatively. The only way to conduct exact quantitative tract tracing is to trace individual neurons, which is demanding and mostly suited to investigate detailed axonal trajectories. Since the purpose of our study was to detect whether topographical distributions were grossly altered in mutant mice, the opted for system level tracing of projections from neuronal populations, which is difficult quantify. Our study involves data from 34 tract tracing experiments (13 mutants, 10 controls and 11 wild types). Following tracer injections in motor areas M1 or M2 of 7 mutants (3 shown in new Fig. 6) we observe similar tracer distribution patterns as in controls, while in 5 out of 6 tracer injections in the somatosensory cortex of mutants (all 6 shown in Fig. 7) yielded highly different labelling patterns compared to controls. We are convinced that these observations warrant the conclusion that topographical distributions are altered in somatosensory but not in motor projections of Nex-cKO mice.*
2. The title somehow contradicts the experimental results, which are mainly obtained from mutant precursors. The abstract needs revision as it does not completely accurately reflect the conclusions that can be obtained from the results.
→***Authors response*:** *We thank the Reviewer for this comment but see that there may be a misunderstanding. The title clearly says that the defect in topographic mapping derives from lack of postmitotic (i.e., neuronal) Nr2f1 expression, and not of precursors. Indeed, while loss* of Nr2f1 in precursor cells leads to an imbalanced ratio between corticopontine and corticospinal (with a higher rate towards the corticopontine population), loss of Nr2f1 in neuronal cells results in topographic shifts that we were able to pinpoint from the S1 subareas and not from motor areas. We think that the title and abstract appropriately reflects these conclusions.

Minor concerns:
In Fig 6, panels E,I and M, the drawings appear to represent elongated images of the corticopontine sections in B-L .
→***Authors response*:** *The images shown in Figure 6E,I,N (now in Fig. 5) may appear elongated since they are shown in perspective, viewed obliquely from ventro-medial. Their spatial proportions have not been altered. It should also be noted that the dot maps only represent signal expression in the pontine nuclei, not signals in the longitudinal fibers of the pons. To better clarify the angle of view for the different panels, we have added several new orientation annotations.*

Reviewer #3 (Significance (Required)):
The brain is a complex network of connections arranged with astounding selectivity and topographic precision but we still do not understand how they are built and to what extent they are driven by intrinsic and extrinsic information. This work aims to understand if Nr2f2 intrinsically determines the connectivity and topography of projections from corticospinal neurons.
Audience: researches in neurodevelopment, neurodevelopmental disorders, modeling of brain networks, neuronal differentiation, and diseases of the nervous system. Expertise: cortical development
→***Authors response*:** *We thank the reviewer for appreciating the importance of understanding intrinsic and extrinsic mechanisms in corticopontine topography and our contribution in this issue.*

© 2022. Published by The Company of Biologists under the terms of the Creative Commons Attribution License (https://creativecommons.org/licenses/by/4.0/).

Referee Cross-commenting

Dear all, I also agree with your reviews and comments.

FOR REVIEWERS ONLY: Pipeline for the automatic quantification of YFP-positive cortical neurons

We propose to perform this analysis by taking advantage of an ImageJ macro that allows automatic counting of spots by area of interest in the brain. The spots are considered as specific staining using a threshold based on intensity and shape of the elements and the composite RGB segmentation atlas as a mask for the regions of interest.

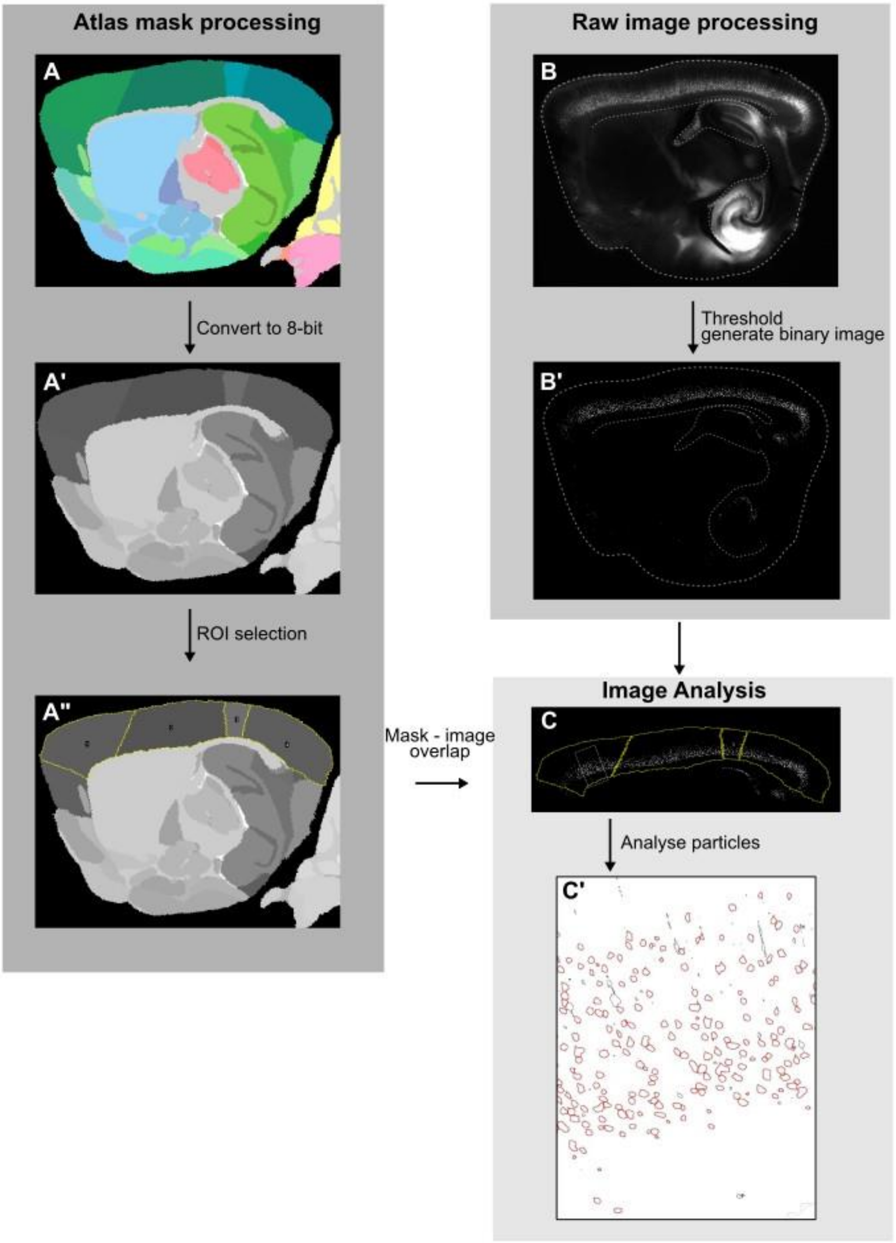

Preliminary steps: create 3 separate folders, one containing the atlas masks (**Panel A**), a second containing the raw TIFF data (**Panel B**) and finally a third folder that will collect all the output images and excel files.

© 2022. Published by The Company of Biologists under the terms of the Creative Commons Attribution License (https://creativecommons.org/licenses/by/4.0/).

Macro steps: from here onwards, every phase is automatically applied to each pair of segmentation mask/raw file.

1. Resizing of the atlas mask to the same size (dimension) as the raw TIFF image, bilinear interpolation.
2. Superimposing of the resized mask to the raw TIFF image
3. Conversion of the resized mask image to 8-bit greyscale (**Panel A'**)
4. Selection of the areas of interest by thresholding (**Panel A''**) (each area of interest is characterized by a specific and unique grey value, i.e.: motor areas, 93; somatosensory areas, 84; auditory areas, 110 and visual areas 94).
5. Background subtraction on the raw TIFF image to eliminate unspecific signal
6. Thresholding to determine the specific signal and binarize the image (Yen thresholding method, value 58) (**Panel B'**)
7. Application of "Watershed" filter to cut out large, sticky spots, avoids clusters/aggregates of spots
8. For each ROI of interest: counting the number of spots via Analyze Particles with a circularity parameter between 0.5 and 1 (to keep only the roundest spots/cells) and a size between 50 and 1500 pixels^2. All elements outside these criteria are rejected. (**Panel C**)
9. Masks corresponding to the selected features of interest are automatically saved in the output folder, under the name "XCountingY", where X is the rank of the analyzed image and Y is the ROI number.
10. A summary Excel file with the 4 ROIs analyzed for each image is automatically saved in the same folder.

References:

Alfano, C., Magrinelli, E., Harb, K., Hevner, R. F. and Studer, M. (2014a). Postmitotic control of sensory area specification during neocortical development. *Nature communications* **5**, 5632.

Alfano, C., Magrinelli, E., Harb, K. and Studer, M. (2014c). The nuclear receptors COUP-TF: a long- lasting experience in forebrain assembly. *Cellular and molecular life sciences : CMLS* **71**, 43- 62.

Armentano, M., Chou, S. J., Tomassy, G. S., Leingartner, A., O'Leary, D. D. and Studer, M. (2007a). COUP-TFI regulates the balance of cortical patterning between frontal/motor and sensory areas. *Nature neuroscience* **10**, 1277-1286.

Bertacchi, M., Parisot, J. and Studer, M. (2019). The pleiotropic transcriptional regulator COUP-TFI plays multiple roles in neural development and disease. *Brain Res* **1705**, 75-94.

Bertacchi, M., Romano, A. L., Loubat, A., Tran Mau-Them, F., Willems, M., Faivre, L., Khau van Kien, P., Perrin, L., Devillard, F., Sorlin, A., et al. (2020). NR2F1 regulates regional progenitor dynamics in the mouse neocortex and cortical gyrification in BBSOAS patients. *EMBO J*, e104163.

Gu, Z., Kalambogias, J., Yoshioka, S., Han, W., Li, Z., Kawasawa, Y. I., Pochareddy, S., Li, Z., Liu, F., Xu, X., et al. (2017). Control of species-dependent cortico-motoneuronal connections underlying manual dexterity. *Science* **357**, 400-404.

Harb, K., Magrinelli, E., Nicolas, C. S., Lukianets, N., Frangeul, L., Pietri, M., Sun, T., Sandoz, G., Grammont, F., Jabaudon, D., et al. (2016). Area-specific development of distinct projection neuron subclasses is regulated by postnatal epigenetic modifications. *eLife* **5**.

Leergaard, T. B., Alloway, K. D., Mutic, J. J. and Bjaalie, J. G. (2000a). Three-dimensional topography of corticopontine projections from rat barrel cortex: correlations with corticostriatal organization. *J Neurosci* **20**, 8474-8484.

Leergaard, T. B. and Bjaalie, J. G. (2007). Topography of the complete corticopontine projection: from experiments to principal Maps. *Front Neurosci* **1**, 211-223.

Leergaard, T. B., Lakke, E. A. and Bjaalie, J. G. (1995). Topographical organization in the early postnatal corticopontine projection: a carbocyanine dye and 3-D computer reconstruction study in the rat. *J Comp Neurol* **361**, 77-94.

Leergaard, T. B., Lyngstad, K. A., Thompson, J. H., Taeymans, S., Vos, B. P., De Schutter, E., Bower, J. M. and Bjaalie, J. G. (2000b). Rat somatosensory cerebropontocerebellar

© 2022. Published by The Company of Biologists under the terms of the Creative Commons Attribution License (https://creativecommons.org/licenses/by/4.0/).

pathways: spatial relationships of the somatotopic map of the primary somatosensory cortex are preserved in a three-dimensional clustered pontine map. *J Comp Neurol* **422**, 246-266.

Mihailoff, G. A., Adams, C. E. and Woodward, D. J. (1984). An autoradiographic study of the postnatal development of sensorimotor and visual components of the corticopontine system. *J Comp Neurol* **222**, 116-127.

O'Leary, D. D. and Terashima, T. (1988). Cortical axons branch to multiple subcortical targets by interstitial axon budding: implications for target recognition and "waiting periods". *Neuron* **1**, 901-910.

Porrero, C., Rubio-Garrido, P., Avendano, C. and Clasca, F. (2010). Mapping of fluorescent protein- expressing neurons and axon pathways in adult and developing Thy1-eYFP-H transgenic mice. *Brain Res* **1345**, 59-72.

Tomassy, G. S., De Leonibus, E., Jabaudon, D., Lodato, S., Alfano, C., Mele, A., Macklis, J. D. and Studer, M. (2010). Area-specific temporal control of corticospinal motor neuron differentiation by COUP-TFI. *Proc Natl Acad Sci U S A* **107**, 3576-3581.

Second decision letter

MS ID#: DEVELOP/2021/200026

MS TITLE: Topography of corticopontine projections is controlled by postmitotic expression of the area-mapping gene Nr2f1

AUTHORS: Chiara Tocco, Martin Ovsthus, Jan G Bjaalie, Trygve Brauns Leergaard, and Michele Studer

I have now received all the referees' reports on the above manuscript, and have reached a decision. The referees' comments are appended below, or you can access them online: please go to BenchPressand click on the 'Manuscripts with Decisions' queue in the Author Area.

As you will see, the referees express considerable interest in your work, but have some significant criticisms and recommend a substantial revision of your manuscript before we can consider publication. The referees remain concerned that the data are insufficient to support the conclusions and suggest additional analyses are needed to strengthen your argument. If you are able to revise the manuscript along the lines suggested, which is likely to involve further experiments, I will be happy to receive a revised version of the manuscript. Your revised paper will be re-reviewed by one or more of the original referees, and acceptance of your manuscript will depend on your addressing satisfactorily the reviewers' major concerns.

We are aware that you may be experiencing disruption to the normal running of your lab that make experimental revisions challenging. If it would be helpful, we encourage you to contact us to discuss your revision in greater detail. Please send us a point-by-point response indicating where you are able to address concerns raised (either experimentally or by changes to the text) and where you will not be able to do so within the normal timeframe of a revision. We will then provide further guidance. Please also note that we are happy to extend revision timeframes as necessary.

Please attend to all of the reviewers' comments and ensure that you clearly highlight all changes made in the revised manuscript. Please avoid using 'Tracked changes' in Word files as these are lost in PDF conversion. I should be grateful if you would also provide a point-by-point response detailing how you have dealt with the points raised by the reviewers in the 'Response to Reviewers' box. If you do not agree with any of their criticisms or suggestions please explain clearly why this is so.

© 2022. Published by The Company of Biologists under the terms of the Creative Commons Attribution License (https://creativecommons.org/licenses/by/4.0/).

Reviewer 1

Advance summary and potential significance to field

The study offers a significant advance in our knowledge of how a specific molecular determinant contributes to the specification and organization of corticofugal projections. The authors have also made much of the data available in supplementary Figures and have presented the findings comprehensively. The study represents one of only a handful of such investigations of corticopontine projections in mice, compares in detail to the topography already described in detail in rats, and will be a welcome resource for those studying the development of corticopontine and corticospinal projections in mice. Although the study is very targeted in scope in this regard, the combination of molecular patterning across development and adult anatomical organization means the study will also appeal to a wider audience.

Comments for the author

The author's have extensively addressed almost all my previous concerns. In particular, the inclusion of a more quantitative and less subjective analysis method to access the spatial distribution of YFP expressing neurons was welcome.
Essential Revisions:
1. Remaining issues with Thy1-YFP line:
In relation to Reviewer 1 point 5, which was a main concern for the reviewers, The authors wrote a relatively clear response for this in the response letter e.g: The YFP expression follows the physiological distribution of subcortical layer V projections, with a high density in motor cortex and a gradually decreased distribution in S1 and more caudal areas (Porrero et al., 2010). Since the distribution of layer V neurons changes in the absence of Nr2f1, as previously reported in our studies (Alfano et al., 2014c; Armentano et al., 2007a; Tomassy et al., 2010), the distribution of Thy1-YFP signal will consequently be altered as a read-out of layer V changes.
This is key information that is, however, still not entirely clear within the manuscript results/discussion. I suggest the authors more explicitly state this around lines 162-166. Specifically adding that the YFP line has been shown to 'follow the physiological distribution of subcortical layer V projections' (Porrero et al., 2010). The study hinges on the fact that Thy1-driven YFP fluorescence is induced in cortical populations specifically related to the specificity of their axonal target regions (and that this relationship would not be interrupted/altered in the conditional KO models).
In this regard, Reviewer 2 point 1, also specified: 'The expression of YFP in the cortex is clearly modified in the mutant. Still, it is unclear whether this is due to the absence of layer V neurons, their respecification to other layer types, or merely to differential regulation of the transgene expression in the mutant lines.' I have not seen that these confounds have been fully identified/addressed. I would suggest a mention of these potential confounds and their respective arguments/explanations in the discussion (perhaps in an additional short paragraph around line 424)
Minor comments:
- In general, the figures have been improved and the addition of the injections site data was appreciated. The summary schematics work much better now in their respective Figure panels. However, I am not sure that the summary Figure 8 is utilized to its full potential. Some indication of the effects of the KO models in the style of this simple schematic may be helpful.
- In Figure 7 J and K, for precision's sake, since this is not a top-down view like 7A but is more from the side, the S1 cells in the brain schematic should be moved to be more dorsal (currently they are represented in a region closer to auditory/association cortex)

Reviewer 2

Advance summary and potential significance to field

This manuscript from Chiara Tocco, Martin Ovsthus, and colleagues explores the consequences of inactivating the gene Nr2f1/COUP-TFI in cortical progenitors or post-mitotic neurons on the topography of the cortico-pontine projection. The authors suggest, using the late cortical driver Nex-cre that post-mitotic deletion of the areal mapping gene COUP-TF1 in corticofugal axons

© 2022. Published by The Company of Biologists under the terms of the Creative Commons Attribution License (https://creativecommons.org/licenses/by/4.0/).

disrupts the topography of the pontine projections. However, the mechanistic cause of this disruption is unclear.

Comments for the author

This revised version of the manuscript from Tocco, Ovsthus, and colleagues brings many improvements in the presentation of the figures from the previous version, especially on the organization of the tracing data and the raw images. However, my comments, as well as the other reviewer's comments, were calling for a more substantive revision, with added data to support the main claims of the organization of the cortico-pontine projections in the conditional Nr2f1 mutants, which are proposed in the schemes of figure 5N-P and 7J,K. My main issues are still present in this version of the manuscript:

- It is not possible to conclude that the organization of the emx1 conditional mutant is similar but just denser than the controls from the data presented in figure 5. Tracings done in Emx1cre mutant, asked by reviewer 1 and me, were important here.
- The most important information to support the claim of disorganization in the Nex-cKO is based on only 1 control injection, shown in Fig7F. It is not correct to match together with the experiments from this manuscript the Allen Brain Institute data, as they were done in very different conditions (not the same virus not the same lines, not the same imaging modality). These crucial tracing experiments must be done properly using stereotactic coordinates in adults, and importantly that more injection points in control animals are made in S1.

  Moreover, the projection patterns obtained from 2 very close sites (7F and 7G) in the mutant are completely different, suggesting strong variability between closely related cases. Unlike the author's claim in the rebuttal letter, the raw images of the injection sites confirm that the cortical areas labeled are extensive as expected, which could account for the variability.
- It is likely incorrect to transfer the areal identity from the controls to the mutants in the injection maps:
  the misguided projections in the Nex-cKO could be from the same absolute coordinate as the controls but belonging to a different functional region due to the cortical remapping. Therefore, the interpretation is not that "S1" projections co-localize with "M2" projections, but that the cortical mediolateral gradient of projections to the pontine nucleus shown in Figure 2 is disrupted.
- Since the authors went through great lengths to build registered, segmented 3D maps of the projection clouds in the pontine nucleus, why not use this to produce quantitative statistical data of the spatial distribution of the projection? The manuscript may benefit from only showing selected raw tracing data side by side (as in 7B and C) and forgoing the confusing segmented data that doesn't add much information over the traditional sections, and detract from the main message without statistical analysis.

Based on the images shown, it is quite likely that there are projection errors and remodeling of the topography of the cortico-pontine projections in the nex-cKO. However, the tracing experiments shown are too preliminary and incomplete to support the precise mapping model proposed. Moreover, the Emx1-cre tracing data would be important to have. I find myself unable to support the publication of this manuscript with these incomplete tracing experiments done in sub-optimal conditions.

Second revision

Author response to reviewers' comments

Response to Reviewers point-by-point:

We are happy about the highly positive comments from Reviewer #1, also supported by the previous Reviewer #3. The comments are constructive and feasible to address. We have added more detailed information to the Discussion and improved Figures 7 and 8, as recommended.

Although we are glad that Reviewer #2 appreciated the improved presentation of our results, we disagree that our use of public normal mouse tracing data is inappropriate and that our conclusions are unwarranted. On the contrary, our study is based on an extensive sharing of raw

© 2022. Published by The Company of Biologists under the terms of the Creative Commons Attribution License (https://creativecommons.org/licenses/by/4.0/).

and lower level data, thereby clearly demonstrating variability and possible outliers in the data. In response to the comments from Reviewer #2 we have clarified and expanded our descriptions at several points to better describe the data and provide interpretations that are compatible with our observations.

Below, we address all comments point-by-point, and explain how we have revised and improved our manuscript. We hope that you will find our revised manuscript worthy of publication in Development.

REVIEWER #1

Advance Summary and Potential Significance to Field:
The study offers a significant advance in our knowledge of how a specific molecular determinant contributes to the specification and organization of corticofugal projections. The authors have also made much of the data available in supplementary Figures and have presented the findings comprehensively. The study represents one of only a handful of such investigations of corticopontine projections in mice, compares in detail to the topography already described in detail in rats, and will be a welcome resource for those studying the development of corticopontine and corticospinal projections in mice. Although the study is very targeted in scope in this regard, the combination of molecular patterning across development and adult anatomical organization means the study will also appeal to a wider audience.

Reviewer 1 Comments for the Author:

The author's have extensively addressed almost all my previous concerns. In particular, the inclusion of a more quantitative and less subjective analysis method to access the spatial distribution of YFP expressing neurons was welcome.

Essential Revisions:

1. Remaining issues with Thy1-YFP line:
In relation to Reviewer 1 point 5, which was a main concern for the reviewers, The authors wrote a relatively clear response for this in the response letter e.g: The YFP expression follows the physiological distribution of subcortical layer V projections, with a high density in motor cortex and a gradually decreased distribution in S1 and more caudal areas (Porrero et al., 2010). Since the distribution of layer V neurons changes in the absence of Nr2f1, as previously reported in our studies (Alfano et al., 2014c; Armentano et al., 2007a; Tomassy et al., 2010), the distribution of Thy1-YFP signal will consequently be altered as a read-out of layer V changes.

This is key information that is, however, still not entirely clear within the manuscript results/discussion. I suggest the authors more explicitly state this around lines 162-166. Specifically adding that the YFP line has been shown to 'follow the physiological distribution of subcortical layer V projections' (Porrero et al., 2010). The study hinges on the fact that Thy1- driven YFP fluorescence is induced in cortical populations specifically related to the specificity of their axonal target regions (and that this relationship would not be interrupted/altered in the conditional KO models). In this regard, Reviewer 2 point 1, also specified: 'The expression of YFP in the cortex is clearly modified in the mutant. Still, it is unclear whether this is due to the absence of layer V neurons, their respecification to other layer types, or merely to differential regulation of the transgene expression in the mutant lines.' I have not seen that these confounds have been fully identified/addressed. I would suggest a mention of these potential confounds and their respective arguments/explanations in the discussion (perhaps in an additional short paragraph around line 424).

Author response
We thank the reviewer for these comments. As suggested, we have added a clarification on pages 6-7 explaining that YFP expression follows the physiological distribution of subcortical layer V projections, with a high density in the motor cortex and a gradually decreased distribution in S1 and more caudal areas, as previously described by Porrero et al., 2010. In the discussion, as suggested by the reviewer, we have added on page 15 that indeed YFP fluorescence is induced in

© 2022. Published by The Company of Biologists under the terms of the Creative Commons Attribution License (https://creativecommons.org/licenses/by/4.0/).

cortical populations related to the specificity of their axonal target regions and that this correlation/relationship is not altered in the conditional *KO* models.

Minor comments:
- In general, the figures have been improved and the addition of the injections site data was appreciated. The summary schematics work much better now in their respective Figure panels. However, I am not sure that the summary Figure 8 is utilized to its full potential. Some indication of the effects of the KO models in the style of this simple schematic may be helpful.
- In Figure 7 J and K, for precision's sake, since this is not a top-down view like 7A but is more from the side, the S1 cells in the brain schematic should be moved to be more dorsal (currently they are represented in a region closer to auditory/association cortex)

Author response
We are happy that Reviewer #1 appreciated our improved Figures. We thank the Reviewer for pointing out the misplaced S1 cells in the brain cartoon in Fig. 7J and K, they are indeed placed too dorsal and posterior. We have corrected this in our revised Fig. 7. We also agree that Figure 8 has potential for improvement and have therefore added a panel that schematically illustrates the observed effects in the *Nex-cKO* model.

REVIEWER 2
Advance Summary and Potential Significance to Field:
This manuscript from Chiara Tocco, Martin Ovsthus, and colleagues explores the consequences of inactivating the gene Nr2f1/COUP-TFI in cortical progenitors or post-mitotic neurons on the topography of the cortico-pontine projection. The authors suggest, using the late cortical driver Nex-cre that post-mitotic deletion of the areal mapping gene COUP-TF1 in corticofugal axons disrupts the topography of the pontine projections. However, the mechanistic cause of this disruption is unclear.

Reviewer 2 Comments for the Author:
This revised version of the manuscript from Tocco, Ovsthus, and colleagues brings many improvements in the presentation of the figures from the previous version, especially on the organization of the tracing data and the raw images. However, my comments, as well as the other reviewer's comments, were calling for a more substantive revision, with added data to support the main claims of the organization of the cortico-pontine projections in the conditional Nr2f1 mutants, which are proposed in the schemes of figure 5N-P and 7J,K. My main issues are still present in this version of the manuscript:
-It is not possible to conclude that the organization of the emx1 conditional mutant is similar but just denser than the controls from the data presented in figure 5. Tracings done in Emx1cre mutant, asked by reviewer 1 and me, were important here.

Author response: It should be noted that the images shown in Figure 5 are selected representative examples, while Supplementary Figures 1 and 2 provide extensive documentation of *Emx1-cKO* animals, compared to controls. In particular, there is a high and confusing number of axonal projections in rostrolateral and ventral parts of the pontine nuclei, which normally receive projections from the occipital cortex, and where a weak signal is seen in the controls. Because of this disorganized and chaotic innervation pattern, single injections in different subregions of the motor and somatosensory cortices would just reproduce the disordered connectivity without unveiling any changes in the topographical organization. The main point of this analysis is that the overall patterns of signal expression are predictably different across the three groups of animals. We, therefore, maintain that our decision to conduct tract-tracing in *Nex-cKO* mice was the best approach to pinpointing the cortical origin of the defects. To emphasize our documentation and rationale, we have added reference to the supplementary figures and revised our explanatory text in the Results section, on pages 11-12 (highlighted in red).

-The most important information to support the claim of disorganization in the Nex-cKO is based on only 1 control injection, shown in Fig7F. It is not correct to match together with the experiments from this manuscript the Allen Brain Institute data, as they were done in very different conditions (not the same virus, not the same lines, not the same imaging modality). These crucial tracing experiments must be done properly, using stereotactic coordinates in adults,

© 2022. Published by The Company of Biologists under the terms of the Creative Commons Attribution License (https://creativecommons.org/licenses/by/4.0/).

and importantly that more injection points in control animals are made in S1. Moreover, the projection patterns obtained from 2 very close sites (7F and 7G) in the mutant are completely different, suggesting strong variability between closely related cases. Unlike the author's claim in the rebuttal letter, the raw images of the injection sites confirm that the cortical areas labeled are extensive as expected, which could account for the variability.

Author response: We disagree with the assertion that the normal mouse tract-tracing data shared by the Allen Brain Institute are not relevant controls for our tracing experiments in mutant mice. Contrary to this, we rather find that the labelling patterns observed in our own control mice are remarkably similar to those observed in corresponding experiments from the Allen Institute. This is shown in Fig. 2 and extensively documented in our supplementary Figures 3-5, showing a large number of highly corresponding results following tracer injections (by the Allen Institute and us) in the motor cortex (compare Supplementary Figs. 3and 4). Also, our control experiment with injection in S1 corresponds nicely with Allen Institute projections traced from the S1 face region (compare Supp. Fig. 3I-K with Supp. Fig. 4). Taken together, all tract-tracing in normal mice (Allen Institute) and our control mice show highly consistent patterns of topographical organization.

The results of our tract-tracing experiments tracing in *Nex-cKO* mice are also consistent.

In all experiments where the tracer was injected medially in the frontal cortex of *Nex-cKO* mice (in locations corresponding to the secondary motor cortex in control mice), we observed curved axonal plexuses located in rostral and caudal positions of the pontine nuclei, surrounding an unlabelled central core. The labelling patterns observed are similar to those observed in our controls and wild-type mice from the Allen Brain Atlas (compare Supp. Fig. 5A-F with Supp. Fig. 4A-I and Supp. Fig. 3A-F).

In experiments where the tracer was injected in the parietal cortex of *Nex-cKO* mice, three main patterns of labelling were seen. One with axonal labelling primarily distributed in the central region of the pontine nuclei, seen in cases injected in the lateral part of the pontine nuclei in regions normally receiving projections from regions representing sensory surfaces of the face (Supp. Fig. 5I-L), a second pattern with distinct rostral and caudal labelled plexuses surrounding the central core region of the pontine nuclei (Supp. Fig. 5G-H), and a third showing a mixture of the two first patterns, with multiple curved axonal plexuses located in both central and rostral regions of the pontine nuclei, seen in cases receiving tracer injections in cortical regions corresponding to S1 whisker representations in wild-type mice. With the exception of one case injected in the anterolateral parietal cortex corresponding to the face / perioral representation in wild-type mice (Fig. 7H and Supp. Fig. 5L), all labelling patterns observed in *Nex-cKO* mice with tracer injections in the parietal (S1) cortex were topographically shifted towards medial or rostral relative to the control experiments (compare *Nex-cKO* cases shown in Fig. 7D-G and Supp. Fig. 5G-K with control cases shown in Fig. 6 and Supp. Fig. 3G-K and 4J). In *Nex-cKO* mice the projections were thus shifted from regions of the pontine nuclei normally receiving projections from S1 to regions normally receiving projections from motor areas.

Thus, by first establishing a hitherto new understanding of the normal 3D topography of mouse corticopontine projections, which is very similar to that described earlier in rats (Leergaard and Bjaalie, Front. Neurosci. 1:211-23, 2007), we maintain that we have a solid benchmark to demonstrate alterations in projections observed in Nex-cKO mice. We do, however, realize from the Reviewers appropriate comment to Fig. 7F-G, that our descriptions and discussion of these patterns have been too concise.

To amend this and better describe the consistency of the spatial distribution patterns observed, we have expanded our results descriptions, added references to the Supplementary Figures on page 12, and further added a comment about data variability in the Discussion on page 14. We thank Reviewer #2 for pointing to the apparent variability in the data presented in Fig. 7, and believe that our expanded descriptions have clarified and improved the manuscript.

-It is likely incorrect to transfer the areal identity from the controls to the mutants in the injection maps: the misguided projections in the Nex-cKO could be from the same absolute coordinate as the controls but belonging to a different functional region due to the cortical

© 2022. Published by The Company of Biologists under the terms of the Creative Commons Attribution License (https://creativecommons.org/licenses/by/4.0/).

remapping. Therefore, the interpretation is not that “S1” projections co-localize with “M2” projections, but that the cortical mediolateral gradient of projections to the pontine nucleus shown in Figure 2 is disrupted.

Author response: Since our study is based on spatial location in the cortex and does not include any functional mapping or use of area identity markers to specify identity, we agree with Reviewer #2 that the projections from locations associated with S1 in normal mice, cannot be labelled as being somatosensory in the mutant brains. To amend this, we have rephrased our descriptions of injection sites in the *Nex-cKO* mice to indicate a topographical location or to specifically mention area identity with respect to normal mice.

-Since the authors went through great lengths to build registered, segmented 3D maps of the projection clouds in the pontine nucleus, why not use this to produce quantitative statistical data of the spatial distribution of the projection? The manuscript may benefit from only showing selected raw tracing data side by side (as in 7B and C) and forgoing the confusing segmented data that doesn’t add much information over the traditional sections, and detract from the main message without statistical analysis. Based on the images shown, it is quite likely that there are projection errors and remodeling of the topography of the cortico-pontine projections in the nex-cKO. However, the tracing experiments shown are too preliminary and incomplete to support the precise mapping model proposed. Moreover, the Emx1-cre tracing data would be important to have. I find myself unable to support the publication of this manuscript with these incomplete tracing experiments done in sub-optimal conditions.

Author response: We can agree with Reviewer #2 that statistical projection maps in principle would have been useful to have, however, we chose not to attempt such analyses, since i) the density of signal expression in mutant mice varied considerably; and ii) since quantitative analyses of tract-tracing experiments are very difficult because tracer injection sites can vary in size and give rise to variable amounts of projections, while the density of projections from different cortical locations may also vary considerably. Our questions and characterizations are therefore focused on the analysis of 3D spatial distribution patterns. The tract-tracing and 3D registration methodology used does give rise to some variability across experiments, but as documented in our supplementary data, the overall patterns observed are consistent across all control experiments, as well as in mutants receiving tracer injections in the frontomedial (motor) cortex (Fig. 6; Supp. Fig. 3, Supp. Fig. 4A), while being very different in mutants injected in the parietal (somatosensory) cortex (Fig. 7B-G; Supp. Fig. 5). Overall, we strongly believe that our data document that the topographical organization corticopontine projections are altered in *Nex-cKO* mice.

Finally, while we can understand that Reviewer #2 would prefer to see our data confirmed by tract-tracing in the *Emx-cKO* line and injections in adult brains of both lines, we would like to clarify that all these requested experiments are extremely time-consuming and would need at least 1 supplementary year of work. In addition, our present animal facility will close in December and only re-derived colonies will be able to enter our new facility. We predict that performing all these requested experiments will last more than 18 months (by also taking into account the expansion of the mouse colonies). Certainly, more detailed studies using different molecular level methods are needed to identify the molecular pathways and mechanisms involved in the establishment of corticopontine topography, but this should be a task for subsequent studies that can build on our findings.

© 2022. Published by The Company of Biologists under the terms of the Creative Commons Attribution License (https://creativecommons.org/licenses/by/4.0/).

Third decision letter

MS ID#: DEVELOP/2021/200026

MS TITLE: Topography of corticopontine projections is controlled by postmitotic expression of the area-mapping gene Nr2f1

AUTHORS: Chiara Tocco, Martin Ovsthus, Jan G Bjaalie, Trygve Brauns Leergaard, and Michele Studer

I apologise for the length of time your paper has been in review, not all of the original Review Commons referees have responded to our requests for comments on your revisions. However I have now received comments and have reached a decision. The referees' comments are appended below, or you can access them online: please go to BenchPressand click on the 'Manuscripts with Decisions' queue in the Author Area.

The overall evaluation is positive and we would like to publish a revised manuscript in Development. There is still a concern that some of the experimental data leave open the possibility of other interpretations and that the strength of conclusions is not always warranted. However, I appreciate that the suggested experiments will be very time consuming. Overall, the consensus of referees opinions is that your data make a valuable contribution to the field. I suggest that an appropriate way to address the remaining concerns is to acknowledge these explicitly in the Discussion in a "Limitations of the Study" section. This would allow you to draw together the caveats you already point out and to include a comment, for example, on the lack of Emx1-cre tracing data.

We are aware that you may currently be unable to access the lab to undertake experimental revisions. If it would be helpful, we encourage you to contact us to discuss your revision in greater detail. Please send us a point-by-point response indicating where you are able to address concerns raised (either experimentally or by changes to the text) and where you will not be able to do so within the normal timeframe of a revision. We will then provide further guidance. Please also note that we are happy to extend revision timeframes as necessary.

Reviewer 1

Advance summary and potential significance to field

The study offers a significant advance in our knowledge of how a specific molecular determinant contributes to the specification and organization of corticofugal projections. The authors have also made much of the data available in supplementary Figures and have presented the findings comprehensively. The study represents one of only a handful of such investigations of corticopontine projections in mice, compares in detail to the topography already described in detail in rats, and will be a welcome resource for those studying the development of corticopontine and corticospinal projections in mice. Although the study is very targeted in scope in this regard, the combination of molecular patterning across development and adult anatomical organization means the study will also appeal to a wider audience.

Comments for the author

The authors have addressed my remaining comments.

© 2022. Published by The Company of Biologists under the terms of the Creative Commons Attribution License (https://creativecommons.org/licenses/by/4.0/).

Third revision

Author response to reviewers' comments

This is the reviewer's comment:
"Reviewer 1 Advance summary and potential significance to field
The study offers a significant advance in our knowledge of how a specific molecular determinant contributes to the specification and organization of corticofugal projections. The authors have also made much of the data available in supplementary Figures and have presented the findings comprehensively. The study represents one of only a handful of such investigations of corticopontine projections in mice, compares in detail to the topography already described in detail in rats, and will be a welcome resource for those studying the development of corticopontine and corticospinal projections in mice. Although the study is very targeted in scope in this regard, the combination of molecular patterning across development and adult anatomical organization means the study will also appeal to a wider audience.

Reviewer 1 Comments for the author
The authors have addressed my remaining comments.

Response: We thank Reviewer 1 to have supported this study confirming that our findings can advance our knowledge of how molecular determinants expressed in gradients in the neocortex contribute to the specification and topographic organization of corticofugal projections.

Fourth decision letter

MS ID#: DEVELOP/2021/200026

MS TITLE: Topography of corticopontine projections is controlled by postmitotic expression of the area-mapping gene Nr2f1

AUTHORS: Chiara Tocco, Martin Ovsthus, Jan G Bjaalie, Trygve Brauns Leergaard, and Michele Studer
ARTICLE TYPE: Research Article

Thank you for sending your manuscript to Development through Review Commons.

I am happy to tell you that your manuscript has been accepted for publication in Development, pending our standard ethics checks.

© 2022. Published by The Company of Biologists under the terms of the Creative Commons Attribution License (https://creativecommons.org/licenses/by/4.0/).
